# A Network View on Psychiatric Disorders: Network Clusters of Symptoms as Elementary Syndromes of Psychopathology

**DOI:** 10.1371/journal.pone.0112734

**Published:** 2014-11-26

**Authors:** Rutger Goekoop, Jaap G. Goekoop

**Affiliations:** 1 Department of Mood Disorders, PsyQ Psychomedical Programs, The Hague, The Netherlands; 2 Parnassia Group, The Hague, The Netherlands; 3 Department of Psychiatry, Leiden University Medical Center, Leiden University, Leiden, The Netherlands; University of Texas at Dallas, United States of America

## Abstract

**Introduction:**

The vast number of psychopathological syndromes that can be observed in clinical practice can be described in terms of a limited number of elementary syndromes that are differentially expressed. Previous attempts to identify elementary syndromes have shown limitations that have slowed progress in the taxonomy of psychiatric disorders.

**Aim:**

To examine the ability of network community detection (NCD) to identify elementary syndromes of psychopathology and move beyond the limitations of current classification methods in psychiatry.

**Methods:**

192 patients with unselected mental disorders were tested on the Comprehensive Psychopathological Rating Scale (CPRS). Principal component analysis (PCA) was performed on the bootstrapped correlation matrix of symptom scores to extract the principal component structure (PCS). An undirected and weighted network graph was constructed from the same matrix. Network community structure (NCS) was optimized using a previously published technique.

**Results:**

In the optimal network structure, network clusters showed a 89% match with principal components of psychopathology. Some 6 network clusters were found, including "DEPRESSION", "MANIA", “ANXIETY”, "PSYCHOSIS", "RETARDATION", and "BEHAVIORAL DISORGANIZATION". Network metrics were used to quantify the continuities between the elementary syndromes.

**Conclusion:**

We present the first comprehensive network graph of psychopathology that is free from the biases of previous classifications: a ‘Psychopathology Web’. Clusters within this network represent elementary syndromes that are connected via a limited number of bridge symptoms. Many problems of previous classifications can be overcome by using a network approach to psychopathology.

## Introduction

The term “psychopathology” is reserved for a type of illness that involves an acute disturbance of mental functions, leading to a significant decline in (inter)personal functioning with respect to a previously attained level of functioning. If recognized and treated well, this type of illness is usually temporary (a ‘dip’). In contrast, ‘personality pathology’ refers to a type of illness that involves a failure to develop habits and mental traits that are required for an adequate regulation of these mental functions in the course of life. Personality pathology therefore involves long-term disabilities in (inter)personal functioning that generally began development in early childhood or puberty. The focus of the current paper is on psychopathology. More specifically, it centers on psychopathology as it occurs at a single moment in time in a heterogeneous sample of patients (a cross-sectional analysis). The temporal evolution or outcome of psychopathology is not the focus of the current paper. Below, we will provide a short overview of previous descriptions of psychopathology with their respective merits and weaknesses. After that, we will provide a redefinition of psychopathology in terms of the mutual relationships between individual symptoms of psychopathology as represented in a network graph.

### A short history of psychopathology

The major syndromes of mental illness have successively been described in three global ways. The first definition is associated with the names of the German physicians Ernst Albrecht Von Zeller and Edward Griesinger (1871), who conceived the whole of psychopathology as different manifestations of a single disorder called “Einheitspsychosis” (usually translated as “Unity Psychosis”). This was a time in which the soul was considered to be an undividable whole, hence different afflictions of the soul could only be due to a single underlying disorder and ‘brain disease’ [1]. To explain the heterogeneity of clinical pictures that could nonetheless be observed (the “phenotypes”), Griesinger assumed that these were due to variations in the intensity of the Einheitspsychosis. Thus, he posited a one-dimensional model of psychopathology in which depression (with anxiety and psychomotor retardation) reflected low-intensity Einheitspsychosis, mania with delusions and rage involved intermediate-intensity levels, and ‘madness’ (with disorganization) and various forms of dementia represented high-intensity levels of Einheitspsychosis [1]. The second global concept of psychopathology is a response to this one-dimensional view of psychopathology and is associated with the German psychiatrist Emil Kraepelin (1892). Kraepelin noted that psychopathological states with high-intensity levels of Einheitspsychosis such as ‘dementia praecox’ (nowadays called schizophrenia) could well appear at once, without necessarily being preceded by lower-intensity syndromes such as depression. Hence, a single-parameter view of psychopathology seemed to fall short of an adequate description of psychopathology. Instead, Kraepelin assumed the existence of *multiple* disease entities [2]. Each of these “natural disease entities” was marked by its own distinct etiology and corresponding neuropathology, which worked its way towards a unique phenotypical expression of illness, which was characterized by its own long-term course and outcome [3]. With this division of the soul into several parts came all the potential complexities of a systems science, since the various neuropathologies and etiologies allowed for a large number of possible interactions. At the time, scientists were not ready for such a view. Indeed, Kraepelin himself must have felt some initial perplexity when referring to the “confusing swarm” of psychotic patients that constituted “the Labyrinth of Psychiatry” [3]. Perhaps as an attempt to reduce complexity, Kraepelin assumed strict demarcations between the various etiologies, neurobiological pathologies and phenotypes. Although he later recognized that certain neurobiological changes and personality traits could produce considerable phenotypical overlap, the original Kraepelinian view on psychopathology is one that precludes relationships between psychiatric disorders [3]. This idea of mental disorders as distinct phenotypical expressions has formed the basis of the third Diagnostic and Statistical Manual of Mental Disorders (DSM-III) of the American Psychiatric Association published in 1980 [4]. Since then, new diagnostic categories have been added to provide a more comprehensive description of the plethora of psychiatric disorders that have been discerned in clinical practice. The DSM of today (which has recently seen its 5th update) contains over 300 disease categories, which are still based on the original Kraepelinian view of categories as phenotypically separable mental disorders (e.g. ‘schizophrenia’).

### Limitations of the categorical approach

The ‘categorical view’ has been subjected to serious forms of critique in the past [5]–[8]. Much of this critique has focused on the untenability of strict demarcations between the disease categories. Previous studies of the co-occurrence of symptoms found no evidence for the proposed categories. The strict phenotypical separations (‘zones of rarity’) that were assumed to exist between neighboring categories could not be observed in reality [6], [7]. Even decades before the publication of the DSM-III in 1980, field studies of mental disorders had already gathered enough scientific evidence to suggest that categorical thinking should be replaced by a more continuous view of psychiatric disorders (e.g. [9]). Later studies showed that the presence of any disorder according to the DSM-III increased the odds of having almost any other disorder, supporting the idea of a phenotypical continuum of psychopathological syndromes rather than separate disease entities [5]. The categorical view therefore clearly does not survive empirical testing. Nevertheless, it has remained in place for decades after these discoveries. This has been due to several global reasons. First, proponents of a continuous view of psychopathology were met with serious resistance, since the absence of strict boundaries between syndromes was thought to make articulated clinical decisions next to impossible [9]. Clinical practice requires well-operationalized diagnostic demarcations as a basis for therapeutic interventions, since such decisions influence important aspects of human lives. Many experts therefore feared that diagnostic systems that assumed continuities between syndromes would render clinical interventions unfocused and powerless. Unfortunately, the possibility of defining well-operationalized demarcations for clinical interventions in a real continuum was not considered. Secondly, the publication of the DSM-III in 1980 was the first attempt at defining standardized diagnostic criteria for mental disorders worldwide. As such, it answered a strong need for diagnostic reliability, even if this went at the cost of validity [10]). Attempts to formulate alternative disease descriptions with a better construct validity were answered by a fear of returning to the chaos of the pre-DSM-III area, in which each school of psychiatry formulated its own diagnostic criteria. Even recently, experts have warned of “pushing psychiatry into chaos” if one were to let go of the grip furnished by the diagnostic categories of the DSM [11]. Such conservative forces have acted as a considerable force of resistance to nosological improvements in the past decades. Meanwhile, the categorical system became so deeply entrenched in government policy and clinical practice that attempts to change it have stranded because of a lack of adaptability. Despite such developments, the past decade has seen a growing consensus amongst mental health care experts that the categorical system overshoots its primary function as a diagnostic tool because of its coarse delineations and lack of validity [12]. However, no consensus yet exists with respect to the adoption of a more suitable view of the structure of mental illness. This seems to be due to limitations of the previous alternatives, which will be shortly discussed below.

### Principal Component Analysis

Historically, studies of human disease have involved studies of collections of symptoms that have a tendency to co-occur. The term “syndrome” has been reserved for such collections of symptoms (from Greek “syn” meaning “together”, and “dromos” meaning “course”). Since the 1950s, researchers have used statistical tests to examine the degree to which symptoms of psychopathology actually co-occur to form syndromes. Most of these studies have used principal component analysis (PCA), in which large numbers of symptoms are grouped together based on their tendency to covary within large numbers of individuals [13]. Thus, collections of significantly covarying symptoms are identified (e.g. low mood, concentration difficulties, excessive worrying), which are called ‘principal components’ or ‘dimensions’ (e.g. ‘Depression’). The total number of components that are found in a dataset is called its ‘component structure’. A small set of components (e.g. Depression, Mania, Psychosis, Anxiety) suffices to represent the majority of symptoms in the entire dataset. The intensity with which a particular component is present in an individual patient can be expressed by a single component score, which is usually calculated by summing the scores on all the symptoms that constitute the component. By expressing the whole of symptom activity within a particular patient as a series of component scores (a ‘multidimensional profile’), complex mental states can be given highly compact descriptions with a minimal loss of information. Each component in a multidimensional profile contributes partly to the overall picture of the clinical syndrome [14]. When a component structure is obtained from a dataset derived from an unselected and heterogeneous sample of patients, these components apply to all patients of that population. Hence, they can be considered ‘universal’ components of psychopathology. Like primary colors on a painter's palette, component scores can be mixed to paint the full landscape of psychopathology in all its subtle gradations. Thus, universal principal components can be viewed as ‘elementary’ syndromes of psychopathology. This approach allows a description of psychopathology that is highly parsimonious and based on empirical measurements. By principle, it avoids the selection bias inherent to diagnostic categories and does not involve rigid cutoffs, exactly meeting the main criticisms of DSM categories. This ‘multidimensional’ view of psychopathology is a third way of describing mental disorders, which has been proposed by the psychiatrist and philosopher Karl Jaspers as far back as 1913 [15].

### Principal components of psychopathology: a weak reception

To date, most of the experience with PCA has been gained in the field of personality psychology. Several elementary personality traits have been identified that allow compact descriptions of the complex personalities of individual subjects. The multidimensional view of human personality has greatly improved the validity, reliability, and descriptive power of personality rating scales [16], [17]. The success of multidimensional ratings of personality has resulted in a considerable consensus with respect to a 5-component structure of human personality, which is used worldwide to assess personality structures in healthy subjects and patients (i.e. neuroticism, extraversion, openness to experience, agreeableness and conscientiousness [18]). In contrast to human personality, however, much less consensus exists with respect to a general component structure of psychopathology. Principal components that have been most consistently identified involve “Depression”, “Psychic and/or Somatic Anxiety”, “Mania or Disinhibition”, “Psychosis”, “Hostility”, “Disorganization”, and “Psychomotor retardation” (see Discussion and [14], [19], [20]). Additionally, syndromes have been identified that involve “Obsessions”, “Dissociation”, and “Cognitive” or “Psycho-organic symptoms” [19], [20]. Despite the rather high replicability of many of these syndromes, psychiatrists have been reluctant to adopt a multidimensional view on psychopathology. A major reason for this weak reception has been the fact that all but two studies of the component structure of psychopathology after 1980 have been performed in patient groups or item pools predefined by the categories of the DSM [20], [21]. As a result, almost as many component structures have been published as there are DSM categories, producing the same heterogeneity of mental disorders as provided by the DSM itself. Because of their limited scope, such components did not reach the status of elementary syndromes of psychopathology that are common to all patients with psychopathology (the purpose of such dimensions has primarily been to assess symptom severity within DSM categories). Additionally, previous component structures of psychopathology differ from each other because of methodological differences, the selection of different patient groups and the use of different rating scales to measure psychopathology. Many of these scales did not cover some of the most clinically relevant symptoms, such as post-traumatic symptoms or symptoms of disorganization. Studies that do report the component structure of a comprehensive part of psychopathology in unselected samples of patients are rare [14], [19]. This can partly be explained by the fact that clinicians often fear that a broad profiling of disease phenotypes will overburden either their patients or their staff. As a result of the above, an incomplete picture of the elementary syndromes of psychopathology has emerged, which failed to convince clinicians of its potential usefulness.

### Limitations of principal component analysis

Apart from such practical reasons, a multidimensional view of psychopathology may not have convinced mental health care professionals because of solid theoretical reasons. First, PCA provides insufficient information on the way in which the various symptoms of psychopathology are tied together into coherent syndromes (dimensions). This is unfortunate, since some symptoms seem to be more important in generating symptom dimensions than others. For example, a symptom such as ‘lassitude’ (or fatigue) is known to correlate to many other symptoms of depression (e.g. inner tension, concentration difficulties, pessimistic thoughts, failing memory), whereas ‘failing memory’ correlates to a fewer number of symptoms [22]. Lassitude therefore seems to be more important in producing covariance and promoting the clustering of symptoms into dimensions than memory failure. Similarly, PCA fails to explain why some symptom dimensions as a whole show preferential relationships, e.g. those between Anxiety, Depression and Retardation [22]–[24], or between Obsessive-compulsive and Phobic disorders [25]). In other words, PCA can't explain comorbidity patterns. PCA just generates sets of symptoms without providing an explicit view on the disparate roles that individual symptoms and syndromes may play in generating their mutual associations. Another reason why factor analytic techniques have been criticized is that some of these methods assume that factors are latent variables that are uniquely responsible for the covariance observed between the symptoms. Such an ‘externally imposed’ view of covariance ignores the possibility that symptom scores covary because of the interactions that exist between the symptoms themselves (i.e. a self-organizational view of psychopathological syndromes [26]). This limitation does not apply to PCA, however, since PCA does not assume the presence of latent variables [13]. PCA differs strongly from factor analysis, but the two techniques are often confused [13]. Whereas factor analysis defines an explicit model of latent variables that explain the covariance observed between (sets of) symptoms, PCA does not [13]. Instead, PCA identifies aggregates of variables that explain most of the variance in the individual variables themselves, without the use of any model. Since almost all multidimensional studies of human personality and psychopathology have used PCA, the objection to the use of latent variables is of less concern in these fields. To summarize, the multidimensional view of psychopathology has been weakly received as an alternative to the categorical view on psychopathology due to conceptual errors and a fundamental inability of PCA to provide an accurate description of the relationships between individual symptoms and syndromes. Hence, it seems worthwhile to examine whether techniques other than PCA can provide a more complete picture of the general structure of psychopathology.

### Network theory and psychiatry

In the past decade, advances in physics and the computer sciences have led to the development of new and powerful tools that enable studies of datasets with very large numbers of variables. Modern network science allows an explicit study of the billions of relationships that may exist between millions of variables in a single network model [27]. In such networks, nodes represent variables (e.g. molecules, organelles, cells, neural systems, symptoms, individual people) and links between the nodes represent their mutual connections (e.g. bonds, ties, correlations). Concepts from network theory are currently pervading different fields of biological science from genetics to sociology and the neurosciences [28], [29]. This has led to a transformation of the way in which we think about biological systems. One of the most important findings from such studies is that biological systems are characterized by a so called “Small World” network topology (e.g. an average of only 6 degrees of separation lie between any two persons in this world, hence the term “Small World” [30]). Connections in such networks are unevenly distributed across the nodes. Most nodes have few connections to other nodes, but some nodes have many. Such richly connected nodes are called ‘hubs’. Because of the existence of hubs, information may travel fast from one part of the network to the other, across a highway of interconnected hubs. Hubs interconnect large numbers of nodes and thus contract certain parts of the network into ‘communities’ (also termed ‘clusters’ or ‘modules’) [31]. Network clusters may themselves show preferential connections to other clusters and behave like nodes in a network at a higher level of biological organization. Small World networks are observed at all spatial scale levels of biological organization (‘from molecules to mind and mankind’), hence living systems are said to exhibit a ‘scale free’ network structure [28]. Depending on their position within the network, some nodes (e.g. hubs) turn out to be more important than others in generating local or global connectivity. Network analysis allows for a detailed study of the way in which individual nodes are involved in generating network clusters and in tying the various clusters together into a globally connected network structure.

Recently, the science of networks has been applied to psychiatry [32]–[34]. Network graphs have been generated of the relationships between symptom scores (e.g. correlations) and these relationships have been transformed into network graphs. In such ‘Psychopathology Webs’, psychiatric symptoms turn out to be vastly interconnected. Individual symptoms have disparate roles in maintaining both local and global connectivity. In psychopathology networks, network clusters (syndromes) can be observed that represent collections of densely interacting symptoms. Symptom clusters are interconnected by individual symptoms (‘bridge symptoms’) that form the boundaries between the various basic syndromes of psychiatric disorders. It turns out that such boundaries are neither discrete nor diffuse [34]. Instead, a limited number of bridge symptoms appears to be responsible for most communication between the basic syndromes (network clusters) [35]. These bridge symptoms may be important in describing the continuities between the syndromes and explaining comorbidity rates. Network analysis methods allow for a detailed quantification of the specific roles that individual symptoms play in maintaining both local and global connectivity in Psychopathology Webs. Since both categorical and multidimensional methods lack the ability to provide such information, this attribute makes network theory a promising candidate as a successor to categorical and multidimensional methods of disease description.

### Current study

Previous network studies have examined only parts of the full symptom space of psychopathology, or only part of the full spectrum of psychiatric patients [35]. Most studies either used non-empirical data [34] or psychometric instruments that were biased by categorical thinking in some way or another (e.g. by skipping certain questions of a rating scale if patients fail to score on a minimum of symptoms that are supposed to belong to the same disease category or diagnostic group according to the DSM). Current literature therefore lacks a view on the network structure of a comprehensive part of psychopathology outside of the zone of influence of the DSM. The current study aimed to provide such a view by studying the nature of the continuities between network clusters (syndromes) observed within a comprehensive network of symptoms of psychopathology. Since DSM thinking has so dominated the field of psychiatry, it is very hard to find data outside of its zone of influence. Our study 1. examined the broadest scope of the psychiatric symptom space that is currently available using a single validated questionnaire 2. examined the broadest scope of psychiatric patients so far examined, and 3. used a rating scale that is unbiased by previous disease classifications. The Comprehensive Psychopathological Rating scale (CPRS) [36], [37] was chosen, which measures symptom scores on a large set of psychiatric symptoms (65). Since it was developed outside of the zone of influence of the DSM, it is one of the few validated rating scales that can provide an unbiased view on the relationships between psychiatric symptoms. The CPRS has good psychometric properties in terms of its within-and between-subject reliability measures [36], [37]. The CPRS measures both reported symptoms and observed signs and symptoms. This allows detection of components such as Retardation and Disorganization that would otherwise go unnoticed when using rating scales that only measure reported symptoms (e.g. the SCL-90). To avoid the narrow view on psychopathology that has resulted from studies that examined patient groups that were selected to conform to predefined DSM criteria, we examined a heterogeneous sample of 192 psychiatric patients suffering from some form of psychopathology as defined in the beginning of the Introduction. Thus, we steered clear of any sample selection biases inherent to previous classifications of mental disorders. From this dataset, a network graph was constructed of the correlational relationships between all symptoms of psychopathology: a ‘Psychopathology Web’. To identify clusters of preferentially co-occurring symptoms, we used a previously published procedure to optimize the community structure of weighted networks, by having PCA and network community detection (NCD) inform each other with respect to the optimal network community structure (modularity) of the dataset [38]. In this previous study, we showed that NCD seems to outperform PCA with respect to its ability to detect plausible modules in datasets with relatively small N. Thus, we expected CPRS symptoms to show clear network communities that represent major syndromes of psychopathology. We expected these communities to be valid in the sense that they showed a good match with the principal component structure of the same dataset, as well as principal component structures of psychopathology published in previous studies. Contrary to categorical or multidimensional descriptions, we expected a rich view on the continuities between the major syndromes of psychopathology. Specifically, we examined the Psychopathology Web for the presence of bridge symptoms that connect the various network clusters of psychopathology. The various roles of individual symptoms in maintaining both local and global connectivity in the Psychopathology Web were quantified and key symptoms were identified as potential targets for treatment. Thus, we aimed to illustrate how a network view of psychopathology can move beyond the limitations of previous methods of disease description such as the categorical and multidimensional views. Finally, the added value of the Psychopathology Web for use in clinical practice was discussed.

## Materials and Methods

### Ethics Statement

All patients in this study provided both verbal and written informed consent and were aware that their psychopathology scores were used for research purposes. This procedure was approved by the Ethics Committee of Leiden University Medical Center. No research was conducted outside our country of residence (The Netherlands) or outside of the context of the institutions that contributed to this study (see affiliations).

### Patient group

In a previous study in 1992, a heterogeneous group of 192 Dutch patients suffering from ‘some form of acute mental illness’ as defined in the beginning of the Introduction was selected for a study of the component structure of mental disorders. The major inclusion criterion was the ability to sustain a clinical interview of about 40 minutes. The major exclusion criterion was insufficient mastery of the Dutch language. This group consisted of 40 newly referred outpatients, 47 patients acutely admitted to a closed department, 73 patients admitted to a short-stay treatment department, and 32 patients residing in medium- and long-stay departments of the same psychiatric hospital. A total of 75 men and 117 women participated (mean age  = 43 years, SD = 6.1 years, range  = 18–86 years). This group was an extension of the group of 99 patients that had previously been described in a report on the inter-rater reliability of the Dutch version of the CPRS [37]. Both DSM-diagnoses and CPRS scores were recorded for all patients. Overall, 45 patients were diagnosed according to the DSM-III within the group of schizophrenic and other primary psychotic disorders, 102 patients had affective disorders (both acute and chronic), 33 had anxious, dysthymic or adjustment disorders and 12 patients suffered predominantly from psychopathology associated with a personality disorder.

### Assessment of symptoms

Patients were screened by different raters in different clinical settings to avoid setting- and rater-specific biases in the correlational structure of the dataset. All raters were trained to administer the CPRS. The training involved the rating of three videotaped or live interviews. During each interview, patients were rated both by an independent clinical psychiatrist that was not responsible for treating the patient and by a research psychiatrist who supervised all ratings and participated in the study. Clinical psychiatrists and the research psychiatrist held consensus meetings after each assessment to improve the consistency of item ratings across the entire sample. CPRS item scores ranged between 0 and 6, which is different from the 0 to 3 scoring used conventionally. This was done to increase item variance, which facilitates the extraction of component structures, and to make these scores compatible with the item grading used for intensity rating scales that have been derived from the CPRS (e.g. MADRS [39]). To our knowledge, our study is unique in the adoption of a data collection procedure that conforms to these high standards. The interview covered the time span of the preceding week and took about 40 minutes to complete for each patient. For raw data, see Information S1.

### Data preprocessing

Items 35 (pathological jealousy, no recorded scores) and 55 (specific speech deficits, no recorded scores) were removed from the analysis, yielding a total of 63 items that were subjected to further analysis. Missing values were rare (mean  = 1.2 (0.6%), SD = 1.5 (0.7%) and were replaced by column mean scores. Next, the 63×63 bivariate (Pearson) correlation matrix of the item scores was bootstrapped (resampled with replacement, n = 10.000 iterations [40]) using SPSS 18, in order to provide a more accurate estimation of the correlation coefficients and p values. Subsequent analyses of the component structure and network cluster structure of the psychopathology dataset were performed on this bootstrapped correlation matrix.

### Construction of network graphs

A network graph was constructed from the bootstrapped symmetrical univariate correlation matrix of item scores, with rows and column names referring to items. No threshold was applied to the matrix to allow inclusion of all correlations between CPRS item scores. This matrix was filled with the corresponding correlation coefficients (r) and transformed into an undirected and weighted network graph by means of NodeXL [22]. In this network graph, ‘nodes’ (vertices) refer to items, ‘links’ to significant correlations between the items, and the ‘weights’ of the links to the corresponding correlation coefficients.

### Network community detection

In order to identify network clusters, we used the Wakita-Tsurumi NCD algorithm integrated within NodeXL [23]. This algorithm is a more efficient variant of the Clauset Newman Moore (CNM) algorithm that finds community structure (“cliquishness”) of nodes within networks in a bottom-up manner, “greedily” optimizing on the modularity of the network graph [24]. The optimal network community structure (NCS) is found by iteratively merging individual pairs of nodes into clusters (and these clusters into superclusters and so on), until maximum ‘modularity’ is reached. Modularity is defined as a measure that expresses the quality of the modular structure of a dataset, with modularity defined as the extent to which “internal” connectivity measures of network clusters are higher than those of the surrounding (external) network. Groups of nodes that share a maximum of connections amongst themselves rather than with their surroundings are high modularity clusters (i.e. high-quality clusters). The Wakita-Tsurumi algorithm implemented in NodeXL deviates from the original version by not including the “heuristics” that help network communities grow in a balanced way. For further details, see [23].

### Optimization of the Psychopathology Web

In contrast to computer networks or the internet, links in correlational network graphs are present with a certain probability, or ‘significance’. The p-score of a correlation (or network link) expresses the probability that the correlation is unjustified (i.e. the link is not there). Hence, the smaller p, the higher the chance of a connection being present. The identification of an optimal network community structure (NCS) in correlational network graphs (such as the network graph of the CPRS dataset) involves the identification of a level of probability p for the significance of a link at which the NCS of the network is optimal. A definitive way of defining a p value at which a NCS is optimal has been lacking from the international literature. Recently, however, we have published a procedure to optimize NCS by means of its principal component structure [38]. This procedure attempts to find the level of significance of network links (correlation coefficient or p value) at which the network community structure of the Psychopathology Web shows an optimal fit with one out of a number of candidate principal component structures (e.g. confirmatory 5, 6, 7, 8, 9, and 10 component PCA; component solutions were rotated using the Varimax criterion). The global threshold for the significance of links is gradually raised in the order of increasing significance (i.e. lowly significant links are successively pruned from the network) until the NCS shows an optimal match with a candidate PCS of the dataset. To express this cluster-to-component match, we used a mismatch (dissimilarity) measure that expresses the absolute number of items within a given network cluster that did not match with the items within a given principal component. This absolute number was divided by the maximum possible mismatch score for that comparison (the sum of the component size and cluster size) to produce a relative mismatch score that varied between 0 (total match) and 1 (total mismatch).

In contrast to the Personality Web described previously in [38], raising the global threshold for the significance of a link in the Psychopathology Web caused some nodes with low correlation coefficients to drop off the network before an optimal match was found. Such ‘isolates’ are weakly connected nodes that during pruning lose their last link to the “main connected component” (i.e. the total body of remaining nodes that are all still interlinked). To avoid an unbalanced match between NCSs and PCSs, a new PCA was performed on the dataset each time a node (item) dropped off the network during pruning (thus, this item was not included in both NCD and PCA). The NCS that showed the closest match with any of the candidate PCSs was kept as the optimized NCS. Its corresponding network graph represented the optimized Psychopathology Web.

### Calculating network metrics

To quantify the roles of individual symptoms of the Psychopathology Web in maintaining network connectivity, weighted versions were calculated of the following network metrics for each node [41]: degree (w_D, number of weighted connections per node, or a measure of the local strength or influence of the node), betweenness centrality (w_BS, a measure of the number of weighted shortest paths that passes the node, or a measure of the global influence of the node throughout the network) and closeness centrality (w_CS, a measure of the extent to which a node can be reached by all other nodes in the network via shortest paths, or how close it is to all other nodes in the network) [42]. Since scores on degree and betweenness centrality are not normally distributed and normality was assumed in further statistical analyses, the natural logarithm was taken of their raw scores to derive a log-linear distribution (yielding ln_w_D, ln_w_BS, ln_w_CS). Next, network measures were compared between network clusters (to identify clusters with specific roles in maintaining network connectivity) and between bridge symptoms and core symptoms (to examine the nature of the continuity between the major syndromes of psychopathology). Bridge symptoms and core symptoms were identified by examining all network nodes for cross-cluster connections. As soon as one or more cross-cluster connections were present, a node was classified as a bridge symptom. Else, nodes were classified as core symptoms. In order to compare network measures statistically, we specified a multivariate general linear model (GLM) in which logtransformed and weighted network measures (ln_w_D, ln_w_BS, ln_w_CS) were dependent variables and cluster membership (CLUSTER, 6 possible values) and bridge symptom status (BRIDGE, 2 values: 1 for bridge symptoms, 0 for core symptoms) were fixed factors. To correct for the possibility that bridge symptoms and core symptoms were unequally distributed across clusters (which could bias the effects of CLUSTER or BRIDGE on network measures), we also specified the interaction CLUSTER*BRIDGE to account for this possible confound. Post-hoc tests were performed to examine differences in estimated marginal means for the following contrasts: 1. CLUSTER (comparing mean scores on network measures between clusters) and 2. BRIDGE (comparing means scores between bridge symptoms and core symptoms).

The cluster coefficient was calculated for each node, which is a measure of the extent to which the direct neighbors of that node are interconnected and (hence) tend to form a cluster. Overall network metrics were calculated that included the mean shortest pathlength of the entire graph (i.e. the average number of links that forms the shortest path between any two nodes of the network). Next, a measure was calculated that expresses the degree of ‘Small Worldness’ of the Psychopathology Web. This measure takes the ratio of the mean clustering coefficient (C) and mean shortest path length (L), which is compared between the empirically derived (CPRS) network and a generalized and randomly connected network graph of the same size ([43]. Since Small World networks are non-randomly connected (with large mean clustering coefficients and short mean pathlengths) this ratio is larger for Small World network than for randomly connected graphs of the same size. Hence, if S>1, the graph can be considered to have Small World properties.

## Results

### Results of principal component analyses

The Kaiser-Meyer-Olkin (KMO) score of the dataset prior to bootstrapping was 0.731 indicating reasonable sampling adequacy. Bartlett's test of sphericity showed a Chi square of 4891.5, df = 1953, p<1.0 10E-12, indicating high sphericity of the dataset.

**Figure 1:**
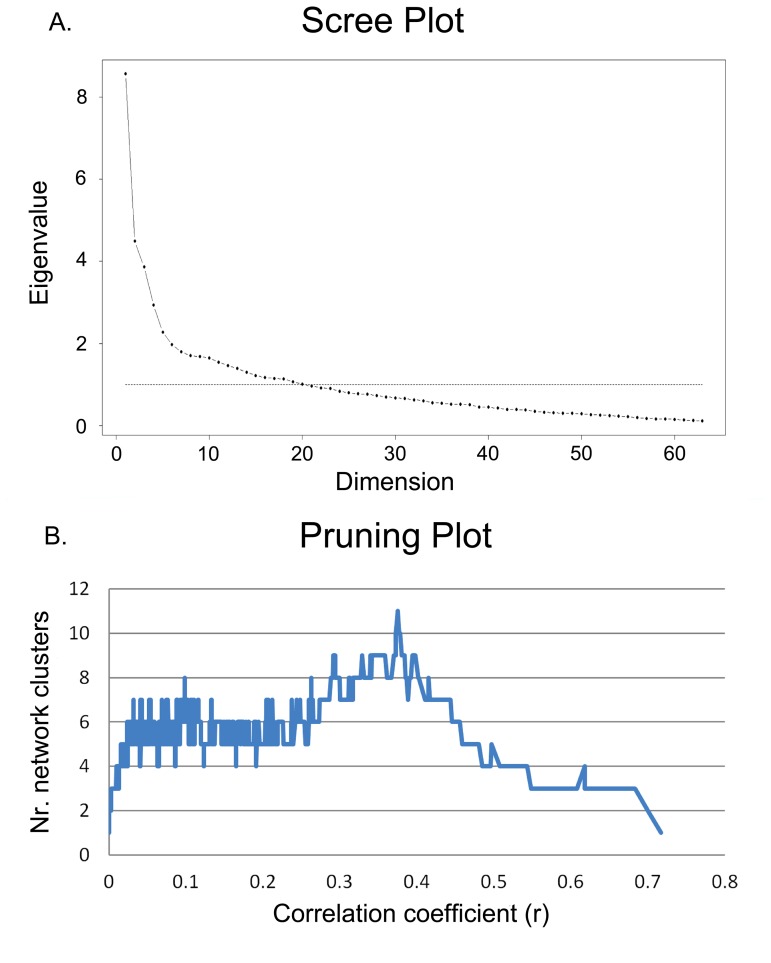
Scree-plot (A) and pruning plot (B) of the CPRS dataset. A. Scree-plot of the exploratory principal component analysis of the CPRS dataset, suggesting a 10-component structure. Defining a cut-off for the total number of components to extract was complicated by the lack of a clear bend in the plot. Hence, 5 additional alternative component structures were matched to the total array of possible network community structures of the dataset, to allow identification of an optimal solution. B. Incremental pruning plot of network community structure analyses showing the number of clusters in the Psychopathology Web as a function of the correlation coefficient that defines the threshold for significance of the links in the network. Neighborless nodes (isolates) are removed from the calculation and do not count as clusters.

Figure 1A shows the screeplot of the PCA performed on the bootstrapped correlation matrix of our dataset. Figure 1B shows the number of clusters resulting from network community structure analyses created during incremental pruning as a function of the correlation coefficient (a ‘pruning plot’). The pruning plot showed that the actual number of clusters in the Psychopathology Web was somewhere between 1 and 11. The screeplot suggested a 10-component structure (10-PCS, data not shown), which deviated from the 5 or 6-PCS found previously in the current dataset without bootstrapping [14]. The lack of a clear bend in the screeplot (Table 1A) made it difficult to establish a clear cut-off for the total number of components to retain from the PCA. Since PCA may produce unreliable results in smaller datasets, 5 additional bootstrapped PCSs (confirmatory 5, 6, 7, 8, and 9 PCSs) were calculated that served as alternative candidate structures. These were matched against the NCSs as summarized in the pruning plot to find a ‘winning’ PCS and NCS. Thus, NCD and PCA informed each other with respect to an optimal solution.

**Table 1:** Table showing the quantitative results of the cluster-to-component matching procedure.

Cluster to component matching			Best Fit			
	Principal Component	% mismatch per cluster	% overall mismatch	ABS(r)	p	nrnodes
5-component structure	PC1	8.6%	43.0%	0.252	4.30E-04	58
	PC2	10.0%				
	PC3	0.0%				
	PC4	23.8%				
	PC5	29.4%				
6-component structure	PC1	14.3%	11.0%	0.268	1.67E-04	55
	PC2	9.1%				
	PC3	0.0%				
	PC4	0.0%				
	PC5	17.6%				
	PC6	0.0%				
7-component structure	PC1	6.7%	27.0%	0.313	1.01E-05	49
	PC2	12.5%				
	PC3	0.0%				
	PC4	0.0%				
	PC5	0.0%				
	PC6	33.3%				
	PC7	42.9%				
8-component structure	PC1	14.3%	43.0%	0.298	2.62E-05	51
	PC2	15.8%				
	PC3	0.0%				
	PC4	0.0%				
	PC5	0.0%				
	PC6	14.3%				
	PC7	71.4%				
	PC8	0.0%				
9-component structure	PC1	18.5%	27.0%	0.294	3.49E-05	52
	PC2	11.1%				
	PC3	0.0%				
	PC4	0.0%				
	PC5	0.0%				
	PC6	14.3%				
	PC7	71.4%				
	PC8	0.0%				
	PC9	20.0%				
10-component structure	PC1	12.0%	31.0%	0.294	3.49E-05	52
	PC2	0.0%				
	PC3	0.0%				
	PC4	11.1%				
	PC5	33.3%				
	PC6	42.9%				
	PC7	11.1%				
	PC8	33.3%				
	PC9	0.0%				
	PC10	66.7%				

Table shows the network community structures (NCS) that were most similar to the confirmatory 5, 6, 7, 8, 9, and 10 principal component structure (PCS) of the CPRS dataset. Component structure: the component structure that was matched against the candidate network community structures obtained from the incremental pruning procedure (see Materials and Methods). Principal Component: the number of the principal component from this component structure. % mismatch per cluster: the percentage of items in a network cluster of the most similar NCS that did not match the item content of its corresponding principal component. % overall mismatch: the percentage of items in the entire NCS that did not match its corresponding PCS. ABS(r): the absolute value of the correlation coefficient at which the optimal match with a NCS was found. p: the corresponding p value. Nrnodes: number of nodes left in the NCS at this threshold (some nodes dropped off the network due to incremental pruning, see text).

### Incremental pruning

As a result of the incremental pruning of network links, individual nodes (symptoms) were marked by unique thresholds (correlation coefficients) at which they lost their final connections with the central connected component of the network. These correlation coefficients (and hence the order of disappearance of nodes or symptom items from the network) correlated positively with the frequency of occurrence of these symptoms (as measured by the percentage of zero intensity symptom scores, r = 0.373, p = 0.003, n = 192). Similarly, component loadings turned out to correlate significantly with frequency of occurrence of the symptoms (r = 0.250, p = 0.048, n = 192). Hence, at least some of the weakly connected items dropped off early during incremental pruning (and showed low component loadings) because of a low frequency of occurrence (this is an inevitable phenomenon in studies of psychopathology, see discussion). Similarly, a trend towards early drop-off was found for items with higher numbers of missing values (r = 0.216, p = 0.089, n = 192) and a similar trend was found for the relationship between component loadings and missing values (r = −0.222, p = 0.081, n = 192). Hence, missing values may partly have contributed to the loss of nodes during incremental pruning.

### Results of network community structure optimization

**Figure 2:**
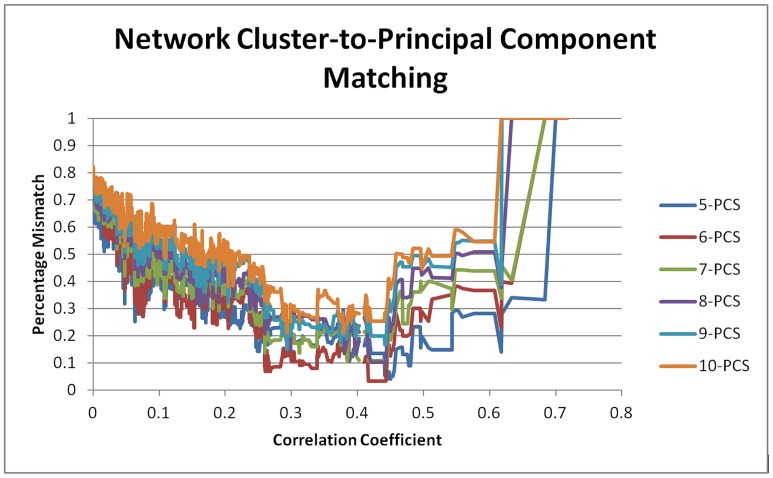
Results of optimizing the network community structure of the CPRS dataset with respect to its principal component structure. Results of the NCS-to-PCS matching procedure for 6 different components structures of the CPRS dataset. X-axis shows the correlation coefficient r as a threshold for significance of a link in the network graph (as r increases to the right, more links are pruned from the network). Y-axis shows dissimilarity (mismatch) scores. Dark blue: 5-PCS, dark red: 6-PCS, green: 7-PCS, purple: 8-PCS, turquoise: 9-PCS, orange: 10-PCS. Mismatch scores collapse at r = 0.27 (p = 1.67E-04), indicating an optimal threshold for the Psychopathology Web and a corresponding six-cluster solution. For details, see text and Tables 1 and 2.

Figure 2 shows the results of matching the full range of network community structures produced by incremental pruning of the Psychopathology Web to 6 alternative component structures (numbered 5–10) of the CPRS dataset. Despite a better global fit of 5-PCS templates, a maximum “local fit” (minimum mismatch scores around a discrete threshold, i.e. a dip) was found with a 6-PCS template. This maximum fit (3.3% mismatch) occurred at r = 0.427, p = 1.80 E-09, after 1904 lesser significant links had been removed from the network (Figure 2). At this threshold, some 35 nodes (isolates) dropped off the network, leaving a total of 28 nodes. At this threshold, the network had fallen apart into several connected components (i.e. percolation occurred). The corresponding 6-component matching template of 28 nodes showed no resemblance to any of the original PCS-templates derived from the whole dataset of 63 items. For these reasons, this ‘maximum’ fit between NCS and PCS was not chosen as an optimal fit with PCS. Instead, the second best fit was chosen, which also involved a 6-component template matching to a 6-network community structure. This second best fit occurred at r = 0.27, p = 1.67E-04, after 1767 lesser significant links had been removed from the network. This unpercolated network consisted of a single connected component, and the original 6-PCS of the unpruned dataset could still be largely recognized in the corresponding 6-component factor structure. This solution was stable for 3 consecutively pruned links (r = 0.2675, to 0.2683, p = 1.75 E-04 to 1.67 E-04). Similar NCS solutions with slightly lesser fits were found across a broad range of correlation coefficients surrounding the optimal threshold (Figure 2). At this threshold, some 8 nodes (isolates) dropped off the network, leaving a total of 55 nodes for the central connected component of the network. NCS and PCA differed with respect to the placement of 5 out of these 55 items (11% mismatch, 89% match). CLUSTER 1 showed a 14.3% mismatch, CLUSTER 2 a 9.1% mismatch and CLUSTER 5 a 17.6% mismatch with their corresponding components (Table 1). The remaining 3 network clusters (clusters 3, 4, 6) showed a complete match (0% mismatch) with their corresponding principal components. Hence, the average mismatch score per component/cluster was 6.8% (SD 8.0%) (Table 1). Table 2 compares the item content of network clusters with that of its matching 6-PCS template.

**Table 2:** Table comparing the component memberships and network cluster memberships of items of the CPRS for the optimal 6-network cluster structure and corresponding 6-component structure.

	PC1	PC2	PC3	PC4	PC5	PC6	CLUSTER1	CLUSTER2	CLUSTER3	CLUSTER4	CLUSTER5	CLUSTER6	
	PSYCHOSIS	ANXIETY	RETARDATION	BEHAV DISORG	DEPRESSION	MANIA	PSYCHOSIS	ANXIETY	RETARDATION	BEHAV DISORG	DEPRESSION	MANIA	Symptom
**it01**					1						1		Sadness
**it02**						1						1	Elation
**it03**					**1**			**1**					**Inner tension**
**it04**		**1**									**1**		**Hostile feelings**
**it05**					1						1		Inability to feel
**it06**					1						1		Pessimistic thoughts
**it07**					1						1		Suicidal thoughts
**it08**													[Hypochondriasis]
**it09**					1						1		Worrying over trifles
**it10**													[Compulsive thoughts]
**it11**					1						1		Phobias
**it12**	1						1						Rituals
**it13**					1						1		Indecision
**it14**					1						1		Lassitude
**it15**					1						1		Fatiguability
**it16**					1						1		Concentration difficulties
**it17**					1						1		Failing memory
**it18**					1						1		Reduced appetite
**it19**		1						1					Reduced sleep
**it20**		1						1					Increased sleep
**it21**					1						1		Reduced sexual interest
**it22**						1						1	Increased sexual interest
**it23**		1						1					Autonomic disturbances
**it24**		1						1					Aches and pains
**it25**					**1**			**1**					**Muscular tension**
**it26**					1						1		Loss of sensation or movement
**it27**					**1**		**1**						**Derealization**
**it28**					**1**		**1**						**Depersonalization**
**it29**	1						1						Feeling controlled
**it30**	1						1						Disrupted thoughts
**it31**	1						1						Ideas of persecution
**it32**						1						1	Ideas of grandeur
**it33**	1						1						Delusional mood
**it34**						1						1	Ecstatic experiences
**it35**													[Morbid jealousy]
**it36**	1						1						Other delusions
**it37**	1						1						Commenting voices
**it38**	1						1						Other auditory hallucinations
**it39**	1						1						Visual hallucinations
**it40**	1						1						Other hallucinations
**it41**					1						1		Apparent sadness
**it42**						1						1	Elated mood
**it43**													[Hostility]
**it44**													[Labile emotional responses]
**it45**			1						1				Lack of appropriate emotion
**it46**		1						1					Autonomic disturbances
**it47**													[Sleepiness]
**it48**				1						1			Distractibility
**it49**			1						1				Withdrawal
**it50**				1						1			Perplexity
**it51**			1						1				Blank spells
**it52**													[Disorientation]
**it53**						1						1	Pressure of speech
**it54**			1						1				Reduced speech
**it55**													[Specific speech defects]
**it56**						1						1	Flight of ideas
**it57**				1						1			Incoherent speech
**it58**				1						1			Perseveration
**it59**						1						1	Overactivity
**it60**			1						1				Slowness of movement
**it61**				1						1			Agitation
**it62**		1						1					Involuntary movements
**it63**		1						1					Muscular tension
**it64**													[Mannerisms and postures]
**it65**													[Hallucinatory behavior]

**Figure 3:**
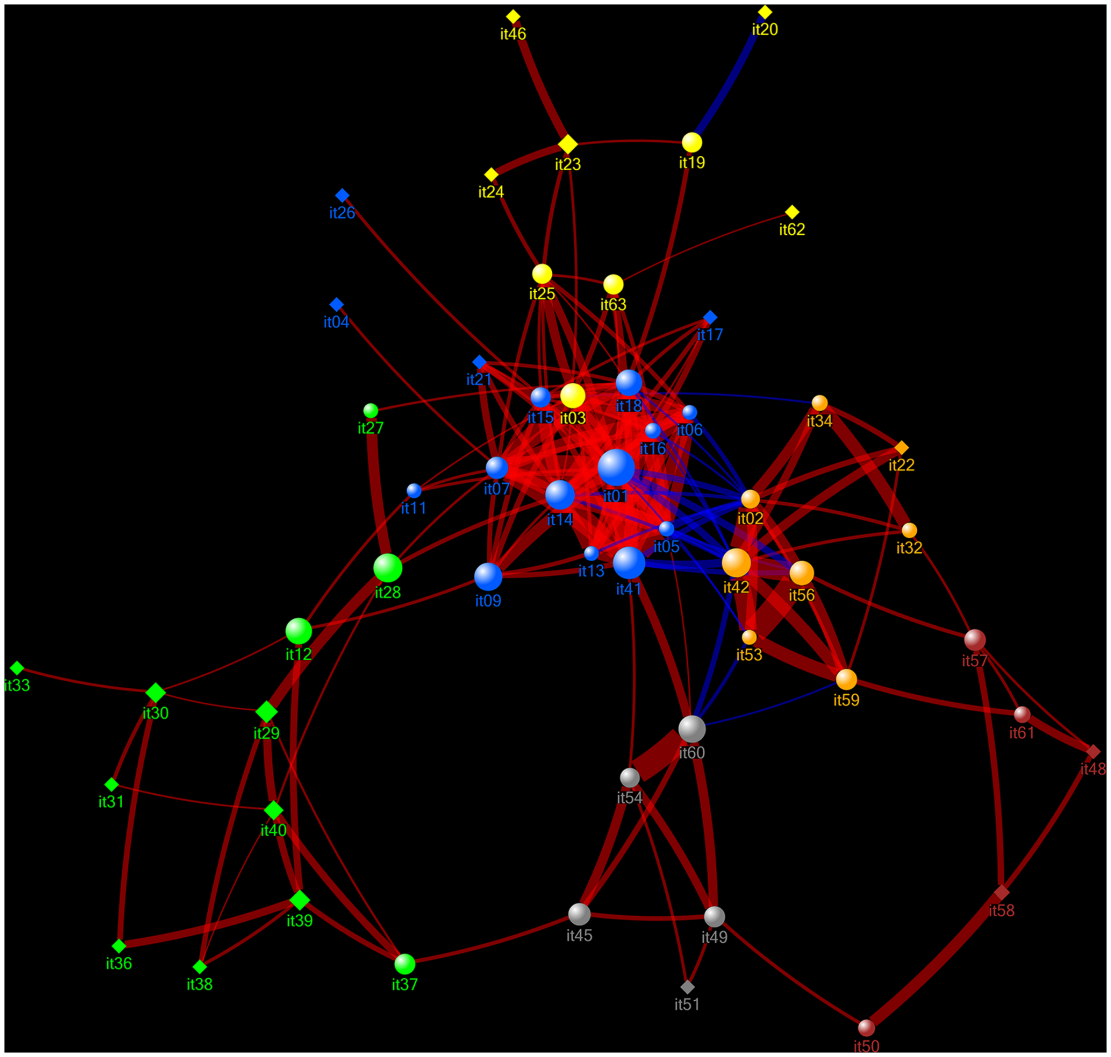
The Psychopathology Web. Network graph of the correlational relationships between 55 items (symptoms) of the CPRS, which form a 6-cluster structure. Node  =  CPRS item (symptom), link  =  significant correlation. The threshold for the significance of network links has been optimized using the procedure described above. Red links: positive correlations. Blue links: negative correlations. The thickness of the links reflects the strength of their corresponding correlation coefficient (weight). It01 etc: item number of the CPRS. Nodes are positioned according to the Hagel-Koren Fast Multiscale layout algorithm. The color of the nodes shows their network cluster membership. Yellow: ANXIETY, Light Blue: DEPRESSION. Orange: MANIA, Green: PSYCHOSIS, Grey: RETARDATION, Brown: BEHAVIORAL DISORGANIZATION. NCD and PCA differ with respect to the placement of items 03, 04, 25, 27, and 28. These mismatches occur at the boundaries between the DEPRESSION cluster and the PSYCHOSIS cluster, and between the DEPRESSION cluster and the ANXIETY cluster and can be interpreted as ‘border disputes’ between NCD and PCA. Spheres: bridge symptoms. Closed diamonds: core symptoms. Node size denotes betweenness centrality score of the node (a measure of its involvement in connecting the various parts of the Psychopathology Web through shortest paths). Smaller and larger loops can be observed that run within and between the various network clusters. See text for further details.

It01 etc: item 1 of the CPRS. Left: matching template of the 6-component structure consisting of 55 nodes. Items were assigned to a single component using a forced-choice filter based on the highest component loadings. Right: the 6-cluster network structure showing an optimal match with the matching template. 1 =  member of this component or cluster, blank  =  not a member. Items that dropped off the network during the incremental pruning procedure or that were discarded from further analyses (it35 and it55, no scores) are shown in [brackets]. Items that were allocated differently by PCA and NCD are shown in **bold.** See also Figure 3.

### The Psychopathology Web

Figure 3 shows the optimized network graph of the CPRS (the Psychopathology Web). With respect to the general topology of the Web, a value of S = 1.94 was found for the Small Worldness measure, indicating that the Psychopathology Web was characterized by a Small World network structure (i.e. network metrics were non-randomly distributed across network nodes). The Psychopathology Web showed a 6-cluster structure. Based on the co-occurrence of items within the network clusters and components and previous findings in the international literature, the following designations were reserved for the network clusters: Cluster 1: DEPRESSION, Cluster 2: PSYCHOSIS, Cluster 3: RETARDATION, Cluster 4: BEHAVIORAL DISORGANIZATION, Cluster 5: ANXIETY, Cluster 6: MANIA. Clusters were interconnected by a limited number of bridge symptoms. In subsequent analyses, we examined how network measures were distributed across individual nodes (symptoms), clusters (syndromes), bridge symptoms and core symptoms.

### Network metrics of nodes and clusters

Table 3 shows network metrics of all symptoms of the Psychopathology Web. Each symptom was marked by unique scores on network metrics, indicating that each symptom in the Psychopathology Web had its own unique role in maintaining network connectivity. Items with above 95% confidence scores for weighted degrees (number of connections) were items 01, 05, 06, 07, 09, 13, 14, 15, 16 18, and 41 (DEPRESSION), items 02, 42, 53, 56, and 59 (MANIA), items 03 and 25 (ANXIETY) and item 60 (RETARDATION). Items with above 95% confidence scores for weighted betweenness centralities were it19 (ANXIETY) items 12, 28, 29, 30, 37, 39, 40 (PSYCHOSIS), items 39, 49, 45, 54 (RETARDATION), items 23 and 63 (ANXIETY) and it57 (BEH DIS). Thus, DEPRESSION and MANIA clusters contained most hubs, whereas PSYCHOSIS, BEH DES and RETARDATION contained most nodes with high betweenness centralities. Since clusters differed considerably in size, differences in the sampling accuracy of individual clusters may have the scores on network metrics of individual symptoms (e.g. the small number of nodes in the RETARDATION or BEH DIS clusters may have limited the possible number of within-cluster connections (degree) of these nodes). Hence, we did not provide a more detailed analysis of the roles of individual symptoms in maintaining network connectivity. Network clusters differed significantly in their mean scores on the combined network metrics of ln_w_D, ln_w_BS, and ln_w_CS (F = 6620, df = 15, p<5.3E -10). These differences mostly involved ln_w_CS (F = 10.861, df = 5, p = 8.50E-7), but not ln_w_D and ln_w_BS. In other words, clusters differed significantly in the ease with which their symptoms could be reached by all other symptoms in the network. Network clusters of psychopathology therefore seemed to have their own roles in maintaining global connectivity. A further specification of the exact roles roles of individual cluster could not be provided due to the low numbers of items in several clusters (e.g. RETARDATION and BEH DIS), which prohibited a reliable comparison of mean scores on network metrics between the various clusters.

**Table 3:** Network metrics of individual symptoms of the Psychopathology Web.

Item	cluster	Bridgeor core	Ext clust	ln_w_degree	ln_w_BS	ln_w_CS
it50	BEH DIS	Bridge	1	1.04	3	−53,700
it57	BEH DIS	Bridge	1	1.75	4	−52,000
it61	BEH DIS	Bridge	1	1.35	3	−53,100
it01	DEPRESSION	Bridge	2	3.48	6	−47,000
it05	DEPRESSION	Bridge	3	3.19	1	−47,700
it06	DEPRESSION	Bridge	2	2.85	0	−48,600
it07	DEPRESSION	Bridge	2	3.11	4	−47,800
it09	DEPRESSION	Bridge	2	2.32	5	−48,700
it11	DEPRESSION	Bridge	2	1.46	0	−49,800
it13	DEPRESSION	Bridge	2	2.62	0	−48,800
it14	DEPRESSION	Bridge	3	3.24	5	−47,400
it15	DEPRESSION	Bridge	1	2.76	4	−48,800
it16	DEPRESSION	Bridge	2	3.01	2	−48,400
it18	DEPRESSION	Bridge	3	3.15	5	−48,100
it41	DEPRESSION	Bridge	3	3.32	5	−47,000
it45	RETARDATION	Bridge	1	1.66	4	−51,000
it49	RETARDATION	Bridge	1	1.87	4	−51,100
it54	RETARDATION	Bridge	1	2.06	4	−49,700
it60	RETARDATION	Bridge	2	2.44	5	−48,900
it02	MANIA	Bridge	1	3.01	4	−48,600
it32	MANIA	Bridge	1	1.64	1	−51,300
it34	MANIA	Bridge	1	1.97	2	−50,300
it42	MANIA	Bridge	2	3.06	5	−48,200
it53	MANIA	Bridge	2	2.44	0	−49,500
it56	MANIA	Bridge	2	2.58	5	−49,200
it59	MANIA	Bridge	2	2.29	4	−50,500
it12	PSYCHOSIS	Bridge	1	1.53	5	−51,100
it27	PSYCHOSIS	Bridge	1	1.00	0	−50,800
it28	PSYCHOSIS	Bridge	1	1.89	5	−49,800
it37	PSYCHOSIS	Bridge	1	1.59	4	−52,500
it03	ANXIETY	Bridge	2	3.11	5	−47,900
it19	ANXIETY	Bridge	1	1.32	4	−51,400
it25	ANXIETY	Bridge	1	2.49	4	−49,500
it63	ANXIETY	Bridge	1	1.95	4	−50,100
it48	BEH DIS	Core	0	1.34	1	−54,400
it58	BEH DIS	Core	0	1.46	3	−53,800
it04	DEPRESSION	Core	0	0.09	0	−51,800
it17	DEPRESSION	Core	0	1.76	0	−50,500
it21	DEPRESSION	Core	0	2.02	0	−50,000
it26	DEPRESSION	Core	0	0.11	0	−52,500
it51	RETARDATION	Core	0	0.80	0	−53,000
it22	MANIA	Core	0	1.65	0	−51,200
it29	PSYCHOSIS	Core	0	1.86	4	−51,500
it30	PSYCHOSIS	Core	0	1.72	4	−53,400
it31	PSYCHOSIS	Core	0	0.78	0	−54,500
it33	PSYCHOSIS	Core	0	0.09	0	−55,900
it36	PSYCHOSIS	Core	0	1.01	1	−54,800
it38	PSYCHOSIS	Core	0	1.22	0	−53,900
it39	PSYCHOSIS	Core	0	1.89	4	−52,600
it40	PSYCHOSIS	Core	0	1.99	4	−51,900
it20	ANXIETY	Core	0	0.36	0	−54,100
it23	ANXIETY	Core	0	1.83	4	−51,400
it24	ANXIETY	Core	0	0.96	0	−52,600
it46	ANXIETY	Core	0	0.41	0	−54,000
it62	ANXIETY	Core	0	0.00	0	−53,600

Item: item of the CPRS. Cluster: name of the network cluster to which the symptom belongs (one of 6 network clusters identified in the CPRS dataset). Bridge or core: specifies whether the symptom is a bridge symptom or a core symptom. Ext. clust.: number of external clusters that the (bridge) symptom connects with. Ln_w_D: logtransformed and weighted degree. Ln_w_BS: logtransformed and weighted betweenness centrality. Ln_w_CS: logtransformed and weighted closeness centrality.

### Network metrics of bridge symptoms and core symptoms

Bridge symptoms were identified that formed direct links between the clusters of the Psychopathology Web. In total, some 34 bridge symptoms were found (Table 3), which is 62% of the total number of nodes in the network. Some 21 symptoms (38%) had only internal connections to their corresponding clusters and were termed ‘core symptoms’ (Table 3). The maximum number of external clusters that was bridged by a single bridge symptom was 3 (Table 3). Network metrics were compared between bridge symptoms and core symptoms. Thus, we were able to perform a detailed analysis of the nature of the continuities between the major syndromes of psychopathology as defined by network clusters. Bridge symptoms showed significantly higher log-transformed and weighted degrees than core symptoms (F = 18564, df = 1, p = 9.5 E-5). Additionally, bridge symptoms had larger log-transformed and weighted within-cluster degrees (mean  = 1.19, SE = 0.08) than core symptoms (mean  = 0.88, SE = 0.13), indicating that the internal connections of bridge symptoms were stronger than those of core symptoms (F = 19225, df = 1, p = 4.2 E-4). Thus, bridge symptoms had a stronger within-cluster influence than core symptoms. Bridge symptoms also showed significantly higher log-transformed and weighted betweenness centralities than core symptoms (F = 19005, df = 1, p = 8.0 E-5). Additionally, bridge symptoms showed significantly higher log-transformed and weighted closeness centralities (mean  = −5.0, SE = 0.021) than core symptoms (mean  = −5.2, SE = 0.033), indicating that bridge symptoms were more easy to reach by all other nodes in the network than core symptoms (F = 37441, df = 1, p = 2.46 E-7). Thus, bridge symptoms had a stronger global influence than core symptoms.

## Discussion

This study presents the first comprehensive network graph of the relationships between symptoms of psychopathology that exist in a heterogeneous group of patients: a ‘Psychopathology Web’. This graph is free of any preconceived conceptual biases of the categorical system of the DSM. It turns out that all symptoms of psychopathology are interconnected. Connections are non-uniformly distributed across symptoms, which causes the Psychopathology Web to contract into several collections of densely interacting symptoms called ‘clusters’. Some 6 network clusters were identified that corresponded to well-known syndromes such as depression, mania, psychosis, anxiety states, inhibited states and behavioral disturbances. Hence, we confirm previous findings of PCA studies that psychopathology has a multimodular structure. Network clusters corresponded strongly to principal components of psychopathology (89%). In contrast to categorical or multidimensional methods, network analysis allows for a detailed analysis of the relative contributions of distinct symptoms in promoting the local clustering of symptoms into syndromes and in connecting the major syndromes of psychopathology. These findings and their clinical significance will be discussed in more detail below.

### The Psychopathology Web

All symptoms of psychopathology were part of a single ‘connected component’. In other words, a global continuity was found between all symptoms and syndromes of psychopathology, which is a finding that argues against the categorical view of psychopathological syndromes as disconnected phenotypes. Yet within this globally connected structure, network clusters were identified that represented a relative autonomy (or segregation) of symptoms with respect to other clusters and the network structure at large. It therefore seems that proponents of segregated psychopathological syndromes and those that favor a more integrated view can reconcile their views by showing that they may have emphasized different aspects of the same multimodular network structure. Below, we will compare the network clusters that were found in the current study with principal components of psychopathology that were identified in previous studies and with related categories of the DSM-IV-TR. Next, we will discuss the benefits of a network view on psychopathology when compared to multidimensional and categorical systems.

### Elementary syndromes of psychopathology

We identified six network clusters that received the following designations based on a review of the multidimensional literature: DEPRESSION, MANIA, PSYCHOSIS, ANXIETY, RETARDATION and BEHAVIORAL DISORGANIZATION. For a more detailed characterization of each of these clusters (and for a comparison of the symptom content of network clusters to that of DSM categories and principal components of psychopathology reported in previous studies), we refer to Information S2. To summarize, the symptom content of the network clusters was highly similar to that of principal components reported in previous studies. Since the 1950s, only two studies have reported a component structure of psychopathology in an unselected sample of acutely admitted patients. These studies have found either 10 or 11 components [20], [44] of which some 6 dimensions were largely retrieved in the current study (with previous labels differing somewhat from those used here).

PCA studies of a more narrow selection of patients using the SCL-90 rating scale have reported separate components for (Retarded) Depression [45]–[48], Somatic Anxiety/Somatization [45]–[48], Anger-Hostility/Irritability [45]–[48], Phobic Anxiety [45], [46], [48], Paranoid Psychoticism [45], [48], Obsessive-Compulsive symptoms [45], [48], Functional Impairment [46] and Attention Problems [47] (the latter two being similar to Obsessive Compulsive symptoms). Notably, none of the SCL-90 studies produced separate components for RETARDATION, DISORGANIZATION and MANIA. For MANIA, this is likely due to the fact that the corresponding items are missing in the SCL-90. Additionally, the SCL-90 does not measure observed items, which is known to prevent detection of RETARDATION and DISORGANIZATION components.

In even more narrowly selected groups of patients, the total number of extracted principal components is typically reduced (see Information S2). which is likely to be the effect of the narrow selection of rated items or the narrow definition of the type of psychopathology that had been studied. Nevertheless, some components in narrowly selected studies still show a great deal of conservation across studies (e.g. Depression, Anxiety, Psychosis) even though differences between rating scales, patient selection procedures, and analysis methods increasingly distort the underlying component structures. Overall, the six clusters reported in the current study turn out to be the ‘usual suspects’ that keep re-emerging in previously published principal component structures in a way that is relatively independent from the subset of patients that is examined or the type of rating scale that is used. We were unable to identify network clusters similar to previously published principal components that contained symptoms of Anger, Obsessive-Compulsive symptoms, Phobias, Organicity, Drug Abuse, Auditory Hallucinations, Depressive Delusions and personality-related clusters. The absence of such clusters can be due to several reasons, which grossly involve psychometric issues (e.g. differences in the type of items included in the various rating scales) and methodological issues (e.g. the patient population that was studied, or the clustering technique that was used). These reasons are discussed in more detail in the Information S2.

Apart from psychometric or methodological issues, the observation that certain clinical pictures cannot be mapped to a single principal component or network cluster may have a more fundamental reason. Multidimensional studies have long shown that psychiatric disorders do not represent unitary syndromes at all, but rather result from the recombination of a limited number of principal components. When such components are extracted from large and unselected patient samples, they are to some degree common to all patients in the sample and can hence be considered to represent ‘universal’ components of psychopathology. Most crucially, however, since the same syndromes can be a part of many different clinical pictures, they can be considered as ‘elementary’ components of psychopathology. For instance, schizophrenia is known to constitute the combined activity of the components of Psychosis (Positive symptoms), Retardation (Negative symptoms) and Disorganization (of behavior and thoughts), with the possible addition of affective components such as Mania or Depression [49]–[51]. Similarly, unipolar melancholic depression according to DSM-IV-TR involves the combined activity of Depression, Retardation and Anxiety [24]. Other subtypes of unipolar depression may be obtained through the admixture of several additional components, such as Psychosis or Anger [52]. Bipolar disorders involve the successive scoring on Depression and Mania components (with low scores on Retardation and high scores on Disorganization). Patients with cyclothymia, hypomania, or mania show differential activity in Mania, Anger, Depression and Retardation components, producing combinations of symptoms that differ in their intensity and severity [53], [54]. Mixed-type bipolar patients may show simultaneous activity within Depression, Mania, Anger, Anxiety and Retardation components [52], [55]–[57]. Schizoaffective disorders represent the (de)synchronized activity of affective components (e.g. Depression, Mania, Anger, Anxiety) and a Psychosis component [58], [59]. Similarly, catatonia represents the combined activity of the Retardation component (e.g. slowness of movement or waxy flexibility), along with Anxiety (high arousal and muscle tension) and Disorganization components (e.g. agitation, perplexity, perseveration) [60], [61]. In all these disorders, the same basic syndromes reappear in different configurations. Thus, the full landscape of psychopathology can be explained by the activity of a limited number of elementary syndromes (typically about 10).

### Elementary syndromes versus previous classifications of psychopathology

With the concept of elementary syndromes in mind, the central problem of the categorical view starts to become more clear. The DSM attempts to provide a list of all possible combinations of elementary syndromes and considers each recombination to be a distinct disease entity in its own right. Previous versions of the DSM have therefore attempted to tackle a combinatorial problem by increasing the number of disease entities, which is an impractical solution. As such, the DSM is much like a list of all colors you can possibly discern, or an inventory of all forms of matter you can possibly encounter, whereas it should actually be more like a list of the primary colors that explain the subtleties in all observable colors through their relative admixtures, or the periodic system of elements in chemistry, which explains all variance in observable matter in terms of the recombination of a limited number of chemical elements. Similarly, the DSM should list all elementary syndromes (primary colors, elements) and provide epidemiological data on the most relevant combinations of these syndromes (color blends, forms of matter). Of course, the most prevalent or relevant of combinations of elementary syndromes can still be given unique names for ease of designation (e.g. ‘retarded-disorganized psychosis’, ‘vegetative-retarded depression’ or ‘vegetative anxiety syndrome’).

Another problem of the DSM is that is has attempted to capture the multitude of causal factors that may contribute to psychiatric disorders into a multitude of distinct disease entities. For example, the DSM recognizes the existence of a “Mood Disorder due to substance abuse”, a “Psychiatric Disorder due to a physical condition”, or a “Psychiatric Disorder due to relational problems”. Thus, previous versions of the DSM have attempted to solve the problem of multifactorial etiologies of psychiatric disorders again by increasing the quantity of disease entities. As a result, the total number of DSM diagnoses has increased even further. Since the number of possible causes of psychiatric illness is near infinite, increasing the number of diagnostic entities is not the solution. One of the most important findings in the past decades has been the fact that the nature of the causal factors that contribute to psychiatric disorders (if known at all) is only relevant to these disorders up to a certain point [62]. The sheer inexhaustible number of biological vulnerabilities, substances and social situations that may afflict the mind ultimately lead to the expression of only 10 elementary syndromes (and about an equal number of basic personality domains). It is as if the brain only has a limited number of global circuits that can be affected by internal or external influences. The total number of discernible syndromes can therefore be drastically reduced, which may significantly add to the clarity of the field.

As a general classification of psychiatric disorders, DSM-5 provides a short list of about 20 main categories. On a first glance, these main categories more closely resemble elementary syndromes (e.g. Depressive Disorders, Bipolar Disorders, Psychotic Disorders). However, such main categories still represent categories that suffer from the limitations discussed above (nonvalid demarcations, combinatorial redundancy and etiological overspecification). For instance, the main category of Addiction according to DSM-5 harbors several categories that contain symptoms referring to withdrawal symptoms. Such symptoms largely involve symptoms of physical Anxiety, which is the same elementary Anxiety component that becomes manifest during panic attacks, claustrophobia or melancholic depressions that are headed under other main categories such as Anxiety Disorders and Depressive Disorders in DSM-5.

From the point of view of elementary syndromes, the problem of current multidimensional research also becomes more clear. With only a few minor exceptions, previous studies invariably examined principal component structures within patient populations or rating scales predefined by the diagnostic categories of the DSM. To use the above analogy, multidimensional research has much been like color specialists attempting to isolate primary colors from narrowly defined color blends such as Orange, Turquoise or Olive Green. As a result, an incomplete view of the elementary syndromes of psychopathology has emerged, which failed to convince clinicians of their potential value. In the same analogy, the use of a heterogeneous sample of patients to extract elementary syndromes is the statistical equivalent of using ‘white light’, which contains a balanced number of all primary colors. The current study is one of the very few studies worldwide that have attempted to provide such a balanced picture. However, we know of no single validated questionnaire that covers all major elementary syndromes of psychopathology and human personality. All current rating scales (including the CPRS) provide an incomplete view of the full landscape of elementary syndromes of psychopathology. We therefore call for the creation and validation of a novel comprehensive rating scale for psychopathology that incorporates all complementary symptoms from previous scales. This scale should be administered in a large and unselected group of patients to extract the full number of elementary components of psychopathology and personality. A large epidemiological survey could then produce the required data on the most prevalent or salient combinations of elementary syndromes and personality domains in different populations of psychiatric patients and the general population.

### Network metrics: not all combinations of symptoms are equally likely

So far, we have shown that network clusters are very similar to principal components and that network clusters of psychopathology qualify as elementary syndromes that can recombine within individual patients to generate a multitude of clinical pictures. This line of thought is not new, nor unique to network theory [15]. Below, we will show that the network approach has additional benefits that are not available using any of the previous methodologies.

Categorical descriptions consider connections between syndromes to be non-existent, whereas multidimensional descriptions underspecify such relationships, or assume that all combinations between components are equally likely. As a result, both techniques are unable to explain preferential pairings between symptoms or syndromes (co-morbidity patterns). In contrast, network analysis allows for a detailed view on the disparate roles that individual symptoms play in generating both local structures such as network clusters, or in connecting the major syndromes of psychopathology into a globally connected whole. The Psychopathology Web turns out to have a Small World topology, which means that connections are unevenly distributed across nodes and clusters throughout the Web. Some (collections of) symptoms are directly connected, whereas others are connected indirectly through a number of intermediate symptoms and connections. This asymmetry in the wiring of symptoms indicates that constraints are put on the likelihood of all possible combinations between symptoms and elementary syndromes. As a result, specific collections of symptoms and syndromes show preferential patters of association (i.e. comorbidity patterns). In a previous (non-empirical) study, Borsboom et al showed that network metrics (preferential connections) between DSM symptoms predicted comorbidity patterns in a realistic manner [34]. Similarly, studies in empirical networks may produce network metrics of bridge or core symptoms that could realistically predict comorbidity patterns. Network analysis allows for a precise quantification of the various roles of individual symptoms and elementary syndromes in generating connectivity across different scale levels of observation and in shaping the general landscape of psychopathology. This will be discussed below.

### Network metrics: defining the local and global influence of symptoms

To examine the local influence of nodes within network structures, a common measure is the weighted degree of these nodes (also called the ‘strength’ or ‘local influence’ of a node). To measure the influence of individual nodes at larger distances, a common measure is weighted betweenness centrality, which is a measure of the number of (weighted) shortest paths that crosses the node, or the degree to which a node is central in mediating traffic between different areas of the network. Finally, a measure of the global influence of symptoms across the network is weighted closeness centrality, which is a measure of the ease with which a particular symptoms can be reached by all other symptoms in the network through weighted shortest paths. In each cluster of the Psychopathology Web, symptoms could be identified with highest weighted degrees and centrality measures for that cluster, indicating that these symptoms played unique roles in generating local (within-syndrome) coherence and/or in knitting the Psychopathology Web together into a globally connected whole (Table 3). A similar analysis was performed for entire network clusters. Overall, symptoms of MANIA and DEPRESSION showed the highest values for both weighted degree and centrality measures (although PSYCHOSIS, BEH DIS and RETARDATION also showed high levels of betweenness centrality). Network clusters differed significantly in their mean weighted closeness centrality, indicating that some clusters as a whole are more easily reached by (closer to) other clusters than others. Together, these results suggest that DEPRESSION and MANIA are the most influential clusters in the Psychopathology Web. This idea is compatible with previous findings that bipolar disorders show high levels of comorbidity with almost all other syndromes of psychopathology such as primary psychotic disorders (schizophrenia), ADHD and anxiety disorders [63]. However, it should be noted that current assessment scales of psychopathology are not specifically designed to measure Psychopathology Webs. Sampling inaccuracies of individual symptoms and clusters may therefore have distorted scores on network metrics. This is a general issue in empirically derived Psychopathology Webs. Since some network clusters were too sparsely sampled to allow reliable comparisons of mean scores on network metrics, we were unable to verify whether indeed MANIA and DEPRESSION clusters showed significant differences in mean influence when compared to other clusters. Nevertheless, since data collection conformed to a number of stringent criteria (see Materials and Methods), we consider the current results to be among the most valid empirical data so far produced on network metrics of psychopathological symptoms. Future studies should aim to capture all relevant elementary syndromes in a single network graph and include many more symptoms per cluster to produce more accurate estimations of network metrics. This would allow a detailed study of the relative importance of specific symptoms and elementary syndromes in generating specific forms of psychopathology.

Our findings somewhat contradict an earlier report of Borsboom et al. that *insomnia* has the highest degree of all symptoms of psychopathology, followed by *psychomotor agitation*, *psychomotor retardation* and *depressed mood* [34]. In our study, insomnia (reduced sleep, it19) rather showed a low degree (as did increased sleep, it20). We do confirm a relatively high degree for psychomotor agitation (agitation: it60) and depressed mood (it01, it41). When compared to the study of Borsboom et al., the CPRS provided a richer sampling of Psychomotor Retardation, which formed a network cluster of its own. Hence, it is difficult to compare network metrics of *retardation* between these two studies. The study of Borsboom et al. also found that symptoms with the highest (random walk) betweenness centrality were *irritable*, *distracted*, *anxious* and *depressed.* In contrast, Irritability (*hostile feelings* (it04) in our study) and *distractibility* (it48 in our study) showed relatively low betweenness centralities in our study. We do confirm relatively high levels of betweenness centrality for symptoms of ANXIETY and DEPRESSION (although the later two formed clusters of their own). It is important to note that the results of Borsboom et al. did not involve direct empirical measurements. Instead, a network was made of DSM-based symptoms, in which two symptoms were connected if they featured within the same DSM category. Given the redundancy of the symptom content of DSM categories (see above), higher degrees or betweenness centralities may have been found in some cases. Also, network metrics in the study of Borsboom et al. were not weighted to account for the strength of the relationships between the symptoms. The above factors may have explain several of the differences with our findings.

### Network metrics: the boundaries between the elementary syndromes

Although power issues prevented reliable statistical comparisons between average scores on network metrics of clusters, we did compare network metrics between bridge symptoms and core symptoms. Bridge symptoms are responsible for connecting the major syndromes of psychopathology using a relatively sparse number of connections [35]. In the current study, we found that bridge symptoms formed about two-thirds of the total number of items in the CPRS, indicating that a majority of symptoms is directly involved in generating continuity between the major syndromes. Roughly one third of items maintained strictly internal connections to items within their own clusters. Bridge symptoms maintained relatively few and weak connections with symptoms outside of their own clusters (bridges), but instead showed large numbers of strong connections to nodes within their own clusters. The internal connections of bridge symptoms were significantly stronger than those of core symptoms, indicating that bridge symptoms are not only responsible for maintaining the external connectivity, but are also mainly responsible for generating the internal connectivity of network clusters. This goes straight against the intuition that bridge symptoms mediate the outward connections of clusters and that core symptoms are responsible for maintaining the internal integrity of network clusters. Additionally, our analyses showed that bridge symptoms had significantly higher values for degree, betweenness and closeness centrality than core symptoms. Hence, bridge symptoms were truly at the center of communication between all parts of the Psychopathology Web. These features make bridge symptoms very well suited to either represent the global states of their own clusters and transmit these states to other clusters, or to receive global states from (bridge symptoms of) other clusters and disseminate such states across their own clusters. Hence, the Psychopathology Web seems to have an architecture that allows clusters (syndromes) to exchange their global states through bridge symptoms. This makes bridge symptoms central players in the development of both psychopathology itself and comorbidity. Since bridge symptoms preferentially connect to specific network clusters, the boundaries between the major syndromes of psychopathology are ‘biased’. Hence, some combinations of syndromes are more likely than others, which can explain observed comorbidity rates. Despite the dominant role of bridge symptoms in the Psychopathology Web, the roles of core symptoms should not be underestimated. Whereas bridge symptoms seem to be mainly responsible for guiding information within and across the various clusters, core symptoms with high degrees and low betweenness centralities may play significant roles in modulating local activity within their own clusters. Indeed, these nodes may 1. represent the majority of all symptoms within their clusters, 2. influence the majority of symptoms within their clusters, or 3. perpetuate activity within their own clusters through positive feedback loops. As such, they may play important roles in the modulation of symptom activity within each elementary syndrome, or in regulating the flow of symptom activity across their own clusters (see below).

### A dynamic view on psychopathology

Psychiatric disorders are dynamical entities. Symptoms and syndromes may come and go and sometimes show intricate mutual dependencies in time such as bipolar or schizotypical disorders. In some cases, the temporal behavior of psychiatric disorders may be their main distinctive feature (i.e. normal cycling and rapid cycling subtypes of bipolar disorders that involve either less or more than 4 episodes of mania in a single year). The importance of making accurate descriptions of the temporal behavior of psychiatric disorders is illustrated by the fact that normal and rapid cycling bipolar disorders respond differentially to treatment [64]. A major benefit of a network view on psychopathology is that it allows a systematic description of the temporal behavior of psychiatric disorders. Central to network theory is the notion that nodes in a network can ‘contaminate’ each other with their states (e.g. inner tension  = > sleep deficits  = > fatigue  = > concentration difficulties) [32]. Thus, symptom scores may travel through the network from node to node across the links in the course of time. This creates a dynamical picture of psychopathology, which is what clinicians observe routinely in their daily practice. Many psychiatric disorders seem to involve ‘vicious cycles’, or feedback loops between symptoms that have a tendency to promote their own existence (e.g. tension  = > sleep deficits  = > fatigue  = > concentration difficulties  = > errors  = > tension, etc). The self-reinforcing character of certain collections of symptoms can in theory be sufficient to bind these symptoms together into syndromes (i.e. a self-organizational view of psychopathology [32], [35]). Self-reinforcing collectives of symptoms can be observed at all spatial scale levels of observation, i.e. at the level of items, subclusters, clusters, or even superclusters. In many cases, psychiatric disorders can be seen as self-referential loops of symptoms that transcend the level of the individual elementary syndrome and encompass several elementary syndromes at once (e.g. ‘schizophrenia’). This dynamical view adds an explanatory power to the study of psychiatric disorders that is unprecedented by previous classifications. Of course, external factors such as environmental or social circumstances may additionally promote covariance between certain sets of symptoms, leaving plenty of room for latent variable hypotheses of psychopathology [65]. Symptom collectives at each spatial scale level are characterized by a unique symptom content (the symptoms that are part of the collective) and temporal scale (i.e. faster or slower collectives). Thus, network science allows a systematic description of both the (state)spatial and temporal aspects of psychiatric disorders. This adds a descriptive and predictive precision to psychiatric nosology that far exceeds that of categorical and multidimensional approaches. In future studies, the results of clinical interventions may be systematically examined with respect to the spatiotemporal features of clinical syndromes.

### External validity of the Psychopathology Web

Most biological networks show a multimodular and scalable network structure, which is the result of their ‘Small World’ architecture [28]. So far, we found evidence that the Psychopathology Web conforms to this general architecture. Additionally, biological networks at all levels of spatial integration show a ‘regulatory’ structure in the sense that specific parts of these networks are dedicated to the representation of the environment (sensing), the valuing of changes in the environment (evaluating), and response formation (acting) based upon these prior states: a perception – evaluation – action loop [29]. When examining the Psychopathology Web for such features, a similar architecture seemed to take shape. The Web has modules dedicated to perception and cognitive evaluation (e.g. the PSYCHOSIS cluster), emotional evaluation (e.g. DEPRESSION, ANXIETY), motivation or conation (MANIA and RETARDATION clusters) and motor action (BEHAVIORAL DISORGANIZATION). To provide more solid evidence of the external validity of network clusters, it is required to link the network structure of the Psychopathology Web to other levels of biological organization (e.g. lower levels of organization such as genomes, proteomes, neurofunctional and structural connectomes, or higher levels of organization such as social networks). It is tempting to speculate that the network structure of Psychopathology at a phenotypical level is partly a reflection of an underlying neural network architecture. Previous neuroimaging studies have indeed shown that hallucinations occur within perceptual areas [66]. Additionally, ANXIETY [67]–[69], DEPRESSION [70], [71] and MANIA [71], [72] are known to involve the major emotional and motivational parts of the brain such as the amygdala and striatum, RETARDATION has been linked to striatal areas that are under dopaminergic control [66] and cognitive and behavioral DISORGANIZATION to attentional and motor- (language) output parts (e.g. [73]). Many previous studies have reported difficulties in linking phenotypic constructs to brain morphology and function [74]. This is at least partly due to the fact that current phenotypical descriptions are ill-defined. We expect that the operationalization of phenotypical constructs in terms of network structures may significantly improve chances of finding neurofunctional and -anatomical correlates of psychopathology. The RDoC initiative specifically aims to integrate biological and phenotypical levels of organization and may well benefit from a network approach [75]. Similarly, the external validity of phenotypic networks can be tested in relationship to higher-level social network structures. Activity within phenotypical networks of individual patients can be related to changes in their topological position within social networks and vice versa. Thus, network science offers a common language and a single conceptual framework from which to explore various levels of biological organization that contribute to psychiatric illness (i.e. biological, phenotypical and social levels) [76]. This facilitates the integration of the various disciplines that study psychiatric patients and avoids difficulties in the interpretation of findings from different fields of study due to the heterogeneity of the methods that are employed.

### Intervening into the Psychopathology Web

A large body of literature shows that Small World networks in general are vulnerable to attacks when nodes that play key roles in maintaining network connectivity (such as bridge symptoms or global hubs) are targeted selectively [77]. As an example, hub nodes can be targeted selectively to disrupt the spread of infectious diseases (e.g. by immunizing high-risk individuals), internet traffic (hacking of key websites) or communication in terrorist networks [78]. Novel network analysis techniques allow identification of a minimal number of influential nodes that can be manipulated selectively to drive the entire network into a desired state. In principle, this allows selective intervention into complex systems [79]. When such findings are translated to the Psychopathology Web, it should be possible to identify several key symptoms or elementary syndromes that could be targeted selectively to promote the dissolution of entire psychopathological states. Although selective targeting is an interesting idea, it is difficult to predict which key nodes are best to target as long as the causal directions of the relationships between these elements are unknown. For instance, global hubs and bridge symptoms can either represent major initiators (‘sources’) or end-stages (‘sinks’) of psychopathology, depending on the amount of causal influences (arrows) that point toward or away from them [80]. Targeting the major sources of psychopathology is likely to be more effective than targeting the sinks. The current study did not focus on causal structures, since the estimation of causal directions is still considered unreliable in networks >30 nodes [81]. Current methods are therefore still unable to accurately identify key symptoms within the Psychopathology Web that allow selective manipulation of psychiatric disorders. Nevertheless, it is possible to examine whether current clinical practice makes sense from a network perspective. Below, we will briefly discuss the effectiveness of some of the more common clinical interventions with respect to several of the key features of the Psychopathology Web.

With respect to pharmacotherapeutic interventions, a limited number of pharmacological classes is available to clinicians. These involve antipsychotics, antidepressants, anxiolytics, mood stabilizers, anticholinergic agents (e.g. to treat psychomotor symptoms that can result from treatment with antipsychotics) and psychostimulants. When examining these global pharmacological domains, there seems to be a considerable overlap with the six main network clusters reported in this paper. Antipsychotics display some degree of preference for the PSYCHOSIS cluster, antidepressants for the DEPRESSION cluster, anxiolytics for the ANXIETY cluster, mood stabilizers for the MANIA cluster, anticholinergic agents for symptoms of RETARDATION (including extrapyramidal side effects of antipsychotic medication) and perhaps psychostimulants for symptoms of BEH DIS. Some agents have especially potent antimanic effects (such as lithium and depakine), whilst other substances are more successful in treating bipolar depression (e.g. lamotrigine or bupropion). Thus, it seems that the elementary syndromes of MANIA and DEPRESSION can be targeted with some selectivity in the treatment of bipolar disorders. Interestingly, no drugs are available that specifically treat symptoms of OCD or dissociation. Such symptoms are often treated with a combination of antidepressants and antipsychotics. Since OCD and dissociative symptoms lie in between the DEPRESSION and PSYCHOSIS clusters (Figure 3, Information S2), this suggests that this ‘neurotic’ subregion of the Psychopathology Web is treated indirectly, by silencing activity in the network clusters that directly surround this region. Hence, a possible function of the Psychopathology Web is to serve as a heuristic tool to identify pharmacological classes that would qualify for the treatment of specific clinical pictures, or to identify a niche for the development of novel pharmacological classes (e.g. anti-compulsive drugs). The existence of multiple elementary domains of psychopathology indicates that combination therapy (i.e. the use of several drug classes with selectivity for certain mental domains) is a more natural way of treating psychopathology than mono-therapy (which is implicated by unitary disease constructs such as disease categories). Indeed, it may be worthwhile for clinicians to examine the main domains of psychopathology that constitute the clinical pictures of individual patients, after which they can systematically review the pharmacological classes that may bring about an optimal reduction of symptom activity within these domains. Similarly, clinicians could use their knowledge of elementary syndromes to systematically screen for side effects of medication, since such effects involve the very same network clusters of the Psychopathology Web (e.g. increased ANXIETY in the first stages of treatment with antidepressants, or RETARDATION as a result of treatment with classical antipsychotics). Thus, current psychopharmacological practice seems to follow the modular nature of psychopathology. A similar trend can be observed in psychotherapy, although psychotherapeutic interventions seem to target elementary modules of human personality rather than psychopathology (e.g. exposure against Avoidance, self-image modules against low Self-directedness, social skills training programs against low Cooperativeness). Considering the above, pharmacotherapy and psychotherapy seem to be directed at a cluster level, although it is possible that certain interventions primarily affect certain key symptoms or personality traits within these clusters. This is an area of future research that needs to examine the selectivity of drugs and other interventions to specific structural features and the temporal behavior of psychopathology and personality webs.

### Potential clinical relevance

We will now briefly summarize the potential benefits of a network view on psychopathology for clinical practice. First, network science enables a new nosology for clinical psychiatry that emphasizes the relationships between symptoms and syndromes and does not presume artificial separations between syndromes (as is the case in the categorical view) or the equality of these relationships (as is the case in the multidimensional view). Despite an increase in complexity on the one hand, this approach generates a huge reduction in complexity by showing how the vast number of psychiatric disorders that can be observed in clinical practice can be described in terms of the interactions between a limited number of modules in the Psychopathology Web. Instead of the 300+ categories of the DSM-IV-TR, clinicians only need to remember a few elementary syndromes. These basic domains can be systematically assessed during history taking and provide a heuristic for clinicians to quickly screen for the presence of the most relevant types of psychopathology or side effects of medication (i.e. triage). Pharmacological treatment strategies could be systemized by decomposing a given clinical picture into its constituent components and systematically reviewing the corresponding medication classes that qualify to treat that particular disorder. This may help clinicians to become more aware of the presence of sequelae or side effects that linger on subclinically (e.g. RETARDATION or DISORGANIZATION) but nonetheless may have a severe impact on the lives of individuals in the long run. Clinicians could be alert to the presence of bridge symptoms, since these could predict the onset of comorbidity and the worsening or spreading of a clinical picture. Additionally, Psychopathology Webs can be used to create risk assessment scales for suicidal tendencies or other symptoms of interest, by examining the network context of symptoms that directly surrounds such symptoms. For instance, it07 (suicidal thoughts) was directly surrounded by reported sadness (it01), apparent sadness (it41), pessimistic thoughts (it06), an inability to feel (it05), inner tension (it03), lassitude (it14), reduced appetite (it08), reduced sexual interest (it21), worrying over trifles (it09), concentration difficulties (it16), fatigability (it15), muscular tension (it25), phobias (it11 (e.g. agoraphobia), hostile feelings (it04), and depersonalization (it28). This symptom context provides a broad view of the psychopathological states that generally govern patients with suicidal thoughts. If patients score on most of these contextual symptoms, clinicians should be alert for suicide attempts. Similarly, clinicians can centralize any symptom of the Psychopathology Web and examine its immediate context, to examine the factors that contribute to these primary complaints. Compressed versions of the CPRS could be developed by selecting the most relevant symptoms within each network cluster (e.g. those symptoms that together explain> 95% of the total cluster score). Such compressed scales can be used to quickly screen patients for the presence of any significant psychopathology or side effects. Compressed scales can significantly facilitate the recording of longitudinal data, since they allow fast yet comprehensive screening of psychopathological symptoms using a minimum of items, requiring less effort from patients. This is very convenient in Routine Outcome Measurement programs or Experience Sampling Methods that measure the effectiveness of clinical interventions. Experience sampling programs can be used to record timeseries that allows assessment of the mutual dependencies between a wide range of mental functions in relationship to a large number of environmental factors. When such longitudinal data are obtained from individual patients, individualized network graphs can be constructed that allow visualization within a single image of the major context factors that contribute to a primary complaint (e.g. a lack of social contacts, lack of exercise, irregular sleep, caffeine use) [82], [83]. Individualized networks can be used in clinical settings as a means of ‘psycho-education’, which helps patients and clinicians to increase their awareness of the reported problems and contributing factors. Such network graphs allow clinicians to formulate custom-tailored interventions that are targeted against the factors that contribute most to the primary complaints of individual patients (personalized medicine). One of the most promising clinical applications of Psychopathology Webs is the possibility to use computer simulations of network activity to predict the future behavior of mental disorders [32]. A recent study has shown that network models of psychopathology allow for the prediction of relapse in major depression for up to three months in advance [84]. Such predictions allow both patients and clinicians to anticipate relapse and take precautionary measures. Finally, it would be interesting to examine how networks of psychopathological symptoms interact with networks of personality traits. Current opinion is that personality is a major context factor for psychopathology and that deficits in specific personality domains are responsible for the occurrence of (different subtypes of) psychopathology [85]–[88]. The occurrence of psychopathology in turn affects scores on personality functions [89], [90]. Personality networks may therefore be important constraints in network simulations that aim to predict the occurrence of psychopathology in individual patients. Additionally, social networks are important determinants of the occurrence of psychopathology. Psychopathology and personality scores affect the positioning of patients in social networks, and vice versa [91]. Network science therefore allows for an integral study of the bio-psycho-social context of psychiatric illness using a single unifying methodology.

### Methodological details and limitations

Although the CPRS clearly does not provide a complete coverage of all known psychiatric symptoms and elementary syndromes, this study was performed under exceptionally stringent data collection criteria (see Materials and Methods). Thus, we believe we provide the most complete and unbiased view of the network structure of psychopathology that is so far available. Multidimensional studies usually require a number of subjects that is six times larger than the number of items in the dataset in order to obtain reliable component structures. In contrast, we previously showed that network cluster algorithms seem to be better than principal component analyses at identifying plausible modules in datasets with relatively small N, since more information is utilized to calculate network clusters [38]. The network clusters in this study showed a strong match (89%) with the principal component structure of the dataset, suggesting good internal validity of the network clusters. Additionally, the network clusters that were detected in our dataset were highly consistent with principal components obtained in previous studies of psychopathology using different patient samples and rating scales. This suggests that the content of the clusters reported in this study was not significantly biased by power issues. Nevertheless, future studies need to examine a broader scope of psychiatric symptoms within a larger number of patients to obtain the most complete and accurate view of elementary syndromes.

The current study employed a previously published method for the optimization of network structures that allowed PCA and NCD to inform each other with respect to an optimal modular structure of the dataset [38]. This technique involved the systematic pruning of weak links from a correlational network graph in the order of increasing connection strength (incremental pruning) until PCA and NCD agreed with respect to an optimal solution. Incremental pruning caused nodes with very weak connections to drop off the network. In a previously published paper on the network structure of human personality according to the NEO-PI-R using the same technique, the loss of nodes during incremental pruning was of less concern, since an optimal match was found before any significant number of nodes dropped off the network [38]. As demonstrated in this study, the greater loss of nodes in Psychopathology Webs when compared to Personality Webs is at least partly due to the presence of lower sampling rates of some symptoms when compared to others. Typically, subjects that are tested on personality questionnaires complete all the items, whereas not all patients suffer from all symptoms on a psychopathology questionnaire. Hence, studies of psychopathology are always biased by symptom frequencies. In PCA studies of psychopathology, more rare items produce lower component loadings that are often reported not to contribute significantly to the component structure. Similarly, nodes that dropped off early in our study as a result of incremental pruning partly represented rare symptoms. Additionally, weak connections may be due to the large heterogeneity of the patient sample. In a group analysis, individual contributions to correlations between item scores are pooled. This may produce considerable variance in the average correlation coefficient between two symptoms (i.e. individual patients are known to differ widely in the correlation coefficients between their symptoms [83]). When a correlation between two items is significant in the Psychopathology Web, this points to the existence of a relationship that on average holds true for the entire group, regardless of its heterogeneity. Such links represent very general relationships within the world of psychopathology. In contrast, the absence of significant correlations between items may either point to the true absence of any relationship between these items, or to a large heterogeneity in the sample with respect to these relationships (e.g. because of the existence of subgroups of patients that differ with respect to these relationships). These problems are inherent to studies of psychopathology at group level and cannot always be corrected for by increasing sample size. Although bootstrapping produced more robust estimates of the individual correlation coefficients, it was unavoidable that subgroup heterogeneity or symptom rarity produced weaker connections. Future studies should focus on gathering large numbers of symptom connectomes from individual patients in a longitudinal setting (e.g. experience sampling methods). Such networks can be pooled to study group-level effects.

The current method of optimizing network structure used correlations to define statistically significant links between symptom scores. However, relationships between symptoms can be defined in many different ways. For instance, partial correlations or regressions may also produce a measure of connectedness. PCA traditionally uses Pearson correlation matrices to identify components. Since the current method relied on PCA to inform network community detection on an optimal solution, we used the same correlation matrix in both NCD and PCA. However, our cluster-to-component matching procedure may work with other matrices and network community detection algorithms as well (see Information S2). Future studies may want to use alternative adjacency matrices and clustering techniques to further improve network community structure optimization.

The Psychopathology Web mostly contained positive correlations between symptoms. Negative correlations were found between several symptoms of MANIA and DEPRESSION, between RETARDATION and MANIA and between increased and decreased sleep in the ANXIETY cluster (Figure 3). Such correlations may represent symptoms that genuinely inhibit each other's presence (e.g. negative feedback loops between MANIA and DEPRESSION). Alternatively, negative correlations may indicate the presence of rare subgroups of patients. For instance, MANIA and DEPRESSION are usually mutually exclusive but may co-occur in rare situations such as mixed-type bipolar disorders. This may explain why the correlation coefficient between symptoms of MANIA and DEPRESSION was not -1 (i.e. related but mutually exclusive), but lies somewhere in between -1 and 0. When examining our data, we found that negative correlations between symptoms of MANIA and DEPRESSION were indeed due to a subgroup of ‘Mixed’ patients that scored simultaneously on symptoms of both MANIA and DEPRESSION. Although some negative correlations may be meaningful, however, this may not be true for all negative correlations. Two variables may show orthogonal scores (i.e. are unrelated), but still produce a significant negative correlation coefficient. This problem can be better understood by plotting the item scores of negatively correlating item pairs in a scatter plot. If two clouds of dots are found that are on or near both axes, the scores are orthogonal and this implies a disconnection. Nevertheless, regression lines try to find a relationship between these data clouds in the form of a line with a negative slope that intersects both the X an Y-axes of the scatter plot. In such a case, negative regression coefficients and correlations are found between these items. Such negative correlations do not represent connections at all, but rather disconnections. We found that negative correlations that are produced in this way tend to be weak (e.g. between increased and decreased sexual interest), and are mostly eliminated by incremental pruning. Nevertheless, future studies need to correct for the presence of (false-positive) negative correlations that are due to the orthogonality of item scores.

In the optimal solution, network clusters and principal components showed a high degree of resemblance (89% at item level). An 11% mismatch was found (5 out of 55 symptoms) that involved the items it03, it04, it25, it27 and it28 (Figure 3, Table 1). Thus, NCD and PCA disagreed with respect to the placement of these nodes into their corresponding modules (clusters or components). These nodes mostly involved ‘bridge symptoms’ that connected two or more network clusters (Figure 3). In reality, bridge symptoms are likely to show ‘fractional’ cluster memberships, i.e. they are partly a member of all the clusters they interconnect. The current method used a forced-choice allocation rule to determine an all-or-nothing membership of a given symptom to its corresponding module (i.e. symptoms needed to declare absolute allegiance to a particular cluster or component in a ‘border dispute’ between the two clustering techniques). Any small differences between the fractional module memberships of bridge symptoms were exaggerated by this procedure. The final cluster membership of such symptoms should therefore not be taken in an absolute sense. When examining the network cluster structure solutions that surrounded the significance threshold for an optimal network structure (r = 0.27), these were very similar to the optimal network structure. The larger bodies of the network clusters remained the same, whereas the allocation of the disputed (bridge) items varied. Thus, the 6-cluster cluster solution presented in the current study was a stable structure, and mismatch scores between component and cluster solutions surrounding the optimal threshold were largely due to the inability of PCA and NCD to agree on the placement of bridge symptoms with ambiguous cluster memberships. In the final solution, one item that was part of an Anxiety component according to PCA (it04, reported hostility) was reallocated by NCD to the DEPRESSION cluster. Two other items that were part of a Depression component according to PCA (it03 (inner tension) and it25 (reported muscular tension)) were allocated to the ANXIETY cluster by NCD. Also, two items that were part of a Depression component according to PCA (it27 (derealization) and it28 (depersonalization)) were moved to the PSYCHOSIS cluster. Most of the disputed items therefore involved the DEPRESSION cluster, which explains the higher mismatch scores for this cluster (Table 1). Thus, (when compared to PCA), NCD produced a more distinct separation of symptoms into groups involving psychological anxiety and physical tension levels (ANXIETY), ‘typical’ depressive symptoms (DEPRESSION) and an altered experience of reality (PSYCHOSIS). Apart from the network clustering algorithm that we used, this may be due to the forced-choice allocation rule employed in the network structure optimization technique.

The nodes of the Psychopathology Web are linguistic constructs that contain some amount of semantic ambiguity (e.g. ‘phobias’) and subjectivity when scored (e.g. ‘observed hostility’). Hence, some level of caution is advised when interpreting network graphs of phenotypical data. It is important to attribute a correct amount of value to the information given by network metrics of nodes and clusters of the Personality Web. However, the CPRS is a very thoroughly studied questionnaire in which redundant questions that explain little additional variance in component scores have been removed. It seems therefore acceptable to regard hub-items in the CPRS as genuine high-degree connectors, and not as the product of badly phrased questions that correlate with many other item scores. The presence of a scalable Small World structure in the CPRS network seems to point in the direction of a biologically plausible network. No prior assumptions were made with respect to the existence of Small World structures so as not to overestimate the information content of our data. Despite such precautions, however, biologically and clinically plausible structures did emerge from the data. As observed, more detailed versions of the Psychopathology Web are required and its external validity should be further examined by comparing its network structure to connectomes at higher and lower levels of biological organization.

## Conclusions

The current article addresses several conceptual issues left by previous taxonomical methods and supplants these with clear and quantifiable concepts derived from network theory. Specifically, a network view on psychopathology seems to solve one of the major nosological issues in psychiatry, which concerns the nature of the boundaries between psychiatric syndromes and the resulting issue of comorbidity patterns. Network models of psychopathology explain the heterogeneity of psychiatric disorders in terms of combinations between elementary syndromes (network clusters) that are differentially expressed across individuals. These syndromes are connected through bridge symptoms, which are highly influential hub symptoms that form the boundaries between the elementary syndromes. It turns out that bridge symptoms are not only disproportionately responsible for interconnecting the elementary syndromes into macroscale psychopathological structures, but also for tying specific sets of symptoms together into elementary syndromes (network clusters). Thus, bridge symptoms are truly at the heart of psychopathology. Since bridge symptoms have preferential connections with specific elementary syndromes, not all combinations of symptoms are equally likely. Thus, psychopathological pictures and comorbidity patterns can be explained in terms of preferential combinations between elementary syndromes that are mediated by bridge symptoms. Apart from explaining local and global aggregations of symptoms in space, the selective connectivity of psychiatric symptoms may uniquely shape the temporal profiles of psychiatric disorders (e.g. in bipolar disorders). Psychopathology Webs can be used to simulate the spread of symptom activity through the network to predict future psychopathological states. Thus, network science adds a descriptive, explanatory and predictive potential to psychiatric nosology that clearly exceeds that of previous classification methods. Since an older doctrine has ruled psychiatry for decades, there is a lack of properly sampled datasets that allow construction of detailed Psychopathology Webs and a full demonstration of the benefits of this new approach. Top priority should be given to the gathering of longitudinal datasets in unselected groups of patients that are broadly sampled at high temporal resolutions with respect to their scores on symptoms of psychopathology, personality traits and social network metrics (i.e. experience sampling methods). A network view on psychopathology opens up a new avenue of clinical applications, several of which are mentioned in the current paper. Overall, scientific methods finally seem to have matured to such an extent as to allow a systems view of psychiatric illness.

## Supporting Information

Information S1 **Raw data file containing item scores that were used to generate the Psychopathology Web.** Scores on items of the CPRS (horizontal) are displayed, as measured in a heterogeneous population of 192 patients with some form of psychopathology as defined in the beginning of the Introduction (vertical). For further information, see Materials and Methods section.
(XLS)

Information S2 **This section provides a characterization of the various network clusters that were identified in the current study, and compares these results to previously reported principal components of psychopathology.**
(DOCX)
