# Supplementary material for: A Network View on Psychiatric Disorders: Network Clusters of Symptoms as Elementary Syndromes of Psychopathology
Source: PLoS One. 2014 Nov 26;9(11):e112734. doi: 10.1371/journal.pone.0112734 (PMC4245101; doi:10.1371/journal.pone.0112734)
Supplement: Information S2 — This section provides a characterization of the various network clusters that were identified in the current study, and compares these results to previously reported principal components of psychopathology. (DOCX) [file pone.0112734.s002.docx]

**SUPPLEMENTARY INFORMATION S2**

In this section, we will provide a characterization of the various network clusters that were identified in the current study, along with a discussion of psychometric and methodological reasons for the differences observed between current network clusters and previously reported components of psychopathology.

DEPRESSION

The DEPRESSION cluster contained symptoms typical of negative affect such as reported sadness (it01), observed sadness (it41) and an inability to feel (it5). This cluster also contained symptoms of reduced motivational intensity such as lassitude (it14), reduced appetite (it18), reduced sexual interest (it21) and indecision (it13), cognitive symptoms such as concentration difficulties (it16) and failing memory (it17) and pessimistic thoughts (it06) (e.g. negative ideas of one’s own capacities). The DEPRESSION cluster also contained anger-related and destructive symptoms such as increased hostility (it04) and suicidal thoughts (it7). These items corresponded highly with the Depression components reported in the only two large studies of the principal component structure of psychopathology so far performed within unselected groups of acutely admitted patients (for a full comparison, we refer to the original studies [1,2]). Like these previous Depression components, the current DEPRESSION cluster strictly contained symptoms related to decreased hedonic functioning and dysphoric mental states. The current DEPRESSION cluster differed mainly from the unselected principal components with respect to the presence of specific neurotic symptoms that occur under the heading of independent categories of the DSM, such as phobias (it11), worrying over trifles (it09), and loss of sensation or movement (it26) (Table 2) (see below).

When including studies in preselected groups of patients, very similar Depression components were found in patients suffering from DSM-type unipolar depression, despite the use of a variety of different rating scales [3-7]. Apart from such Depression components, PCA in unipolar depressed patients yielded several additional components, including Anxiety (e.g. [6-9]), Anger (e.g. [10-12]), Positive Affect / Activation (e.g. [6,13]), Positive (psychotic) symptoms (e.g. [14,15]), negative symptoms / retardation (e.g. [7,14-18]) and Disorganization (e.g. [14,15,19]). When considering component structures of DSM-selected patient groups other than unipolar depression, similar Depression components have been found in patients with mixed anxious-depressive pathology (e.g. [8,20-23]), bipolar disorders (e.g. [24-27]) and patients with schizophrenia and related disorders (e.g. [27-31] (for a detailed comparison between the various components, we refer to the original studies). Thus, similar Depression components were identified in different patient groups using a variety of different rating scales. This supports the presence of a universal syndrome of DEPRESSION. Each rating scale seems to harbor items that are complementary in detecting this syndrome. The differences observed between the various Depression components in these studies can be explained by differences in symptom content between the scales, selection biases introduced by patient selection procedures, low sample sizes and methodological differences (e.g. different rotation procedures during PCA).

In the current study, the DEPRESSION network cluster contained most of the symptoms that are part of the unipolar depression category according to the DSM-IV-TR. Only the items of increased appetite and weight gain were lacking, since these are not measured by the CPRS. Depression components from previous studies also showed considerable correspondence with the unipolar depression category of the DSM. This shows that the DSM category of unipolar depression may be a reasonable estimation of a universal depression syndrome. Nevertheless, the DSM-IV-TR category for unipolar depression does not contain the items of reduced sexual interest (it21) and failing memory (it17), despite the common occurrence of these items in a majority of the Depression components found in previous studies. Future studies should aim to identify a more definitive version of the DEPRESSION syndrome.

MANIA

The network cluster identified as MANIA contained symptoms typical of positive affect such as elation (it02) and elated mood (it42), increased motivational intensity as measured by ‘increased sexual interest’ (it22) and a heightened sense of purpose and meaning as measured by ‘ecstatic experiences’ (it34), accelerated cognitive processing (flight of ideas (it56), positive cognitions about the self (ideas of grandeur (it32)) and increased motor action (pressure of speech (it53), overactivity (it59)). The MANIA cluster was highly similar to the Mania components of previous PCA studies in unselected groups of patients (i.e. “Mania-agitation” [2] and “Mania“ [1]). Like these previous Mania components, the current MANIA cluster strictly contained symptoms related to euphoric manic states and increased hedonic functioning. PCA studies in groups of patients preselected to conform to the DSM criteria of a bipolar I disorder have identified highly similar ‘hedonic’ Mania components, despite the use of different rating scales and the selection of different patient samples (e.g. euphoric or dysphoric patients). These hedonic components have been referred to as “Euphoric Mania” [24], “Elation-Euphoria” [25], “Hedonism” [26], “Increased Hedonic Function” [32] and “Excitement” [27]. Apart from such hedonic components, these studies identified several additional components, including Depression-like components such as Depression in [24-27] or Depression / sexuality in [25], Anger-like components such as Hostility [33], Dysphoria [24,26,32], Irritable Aggression [32], Paranoia-Hostility [25], and Psychosis-like components e.g. Psychosis in [15,24,26,32,33], Grandiosity-Psychosis in [25] or Positive symptoms in [14,15,27]. Separate components have been identified for Psychomotor Acceleration [32]) / Activation [26] (resembling Disorganization syndromes), and Psychomotor Retardation or slowing down (e.g. Deficit in [25], or Negative Symptoms in [14,15,27]). Most studies of bipolar I patients excluded symptoms of anxiety, but when such items were included, separate Anxiety components were found (e.g. [27,33]). In some cases, Anxiety symptoms formed a conjunction with motor hyperactivity to form components such as “Mixety” [24] or Disinibition-Instability [25]. When considering component structures of DSM-selected patient groups other than bipolar I, highly similar hedonic (Mania) components have been found in hypomanic (bipolar II) [34], ‘unipolar’ depressed [6,13], anxious [21] and psychotic patients [14,28] and in the general population [35]. Since the above studies involved different rating scales and patient groups but yielded similar hedonic Mania components, this supports the existence of a universal syndrome for (hedonic) MANIA. Patient selection procedures, the use of different rating scales, methodological and power issues are likely to have produced distorted views of this universal syndrome in the past.

DSM-IV-TR criteria for mania matched very well with the item content of the network cluster identified as MANIA in the current study. Indeed, 7 of the 8 symptoms from the MANIA network cluster corresponded with 6 of the 8 items of the DSM-IV-TR. DSM-5 requires the presence of increased energy / overactivity as a new primary characteristic in bipolar disorders, which occurs frequently as a symptom in principal components of mania and is part of the MANIA network cluster. This again shows that DSM-categories may sometimes represent well-estimated universal syndromes, but that new studies should be performed in large and heterogeneous patient samples using complementary items from all Mania rating scales to isolate the complete universal syndrome.

ANXIETY

The ANXIETY cluster of the current study contains symptoms that involve states of both psychic anxiety (i.e. “inner tension” (it03), which covers symptoms such as ill-defined discomfort, edginess, inner turmoil, mental tension mounting to panic, dread and anguish) and physical anxiety, which is often referred to as ‘somatic anxiety equivalents’, ‘physiological hyperarousal’, and ‘autonomous’ or ‘vegetative’ symptoms. These involves autonomous symptoms (it23 and it46; such as palpitations, breathing difficulties, dizziness, sweating, cold hands and feet, dry mouth, indigestion, diarrhea, frequent micturition), muscular symptoms (it25 and it63, such as increased muscular tension), involuntary movements (it62), aches and pains (it24), and complaints related to the wake-sleep cycle (it19 and it20, i.e. decreased and increased sleep). This cluster contents is almost identical to the Anxiety components of the two previous PCAs in large heterogeneous samples of patients, in which Psychological Anxiety and Somatic Anxiety equivalents both belonged to a single cluster. For an exact comparison, see ‘Anxiety’ in [1] and ‘Anxiety’ in [2]. Little studies exist that have examined principal component structures within circumscript groups of anxious patients as defined by the DSM. In patients with generalized anxiety disorder (GAD) and Panic Disorder (PD), separate components have been found for Psychic Tension and Somatic Tension [36] as well as Depression [8,20]. In patients with post-traumatic stress disorder (PTSD), Hyperarousal (Psychic and/or Somatic Tension) has been systematically identified, along with several components for Dysphoria / Anger and Emotional Numbing [37,38]. Although OCD and Phobias are categorized amongst anxiety disorders in the DSM, PCA studies of these disorders have systematically excluded affective symptoms from these studies (research mainly focused on the objects of fear rather than the fear itself). Not surprisingly therefore, no components of Anger, Anxiety or Depression are reported in such studies. In most cases, PCA studies of anxiety-related states involved mixed groups of both anxious and depressed patients. Such studies have consistently identified separate components for Psychic Anxiety and/or Somatic Anxiety, apart from Depression components [8,20-23]. PCA studies in DSM-selected patient groups other than anxiety disorders have often neglected a systematic examination of anxiety symptoms. Nevertheless, Psychological and/or Somatic Anxiety components have been reported in DSM-selected unipolar depressed [6-9]), bipolar [21,27,33] or psychotic patients [15,28,30,31,39] using a variety of different rating scales. Thus, as soon as anxiety symptoms are included in PCA studies, Somatic and/or Psychological anxiety components appear regardless of the patient groups under study or the type of rating scales that are administered. Somatic and Psychological Anxiety components merge into a single component in unselected patient groups [1,2] and in the current unselected sample. Hence, the existence of separate clusters for somatic and psychological anxiety does not seem to be a universal trait, but rather a consequence of the patient selection procedure that points to the existence of subgroups of patients that differ in the expression of psychic and somatic anxiety. Thus, Anxiety (like Depression an Mania) qualifies as a universal syndrome of psychopathology. Future studies need to examine anxiety states in relation to a broad context of other psychopathological states in order to identify their mutual relationships.

The symptoms of the ANXIETY cluster are separately contained under different diagnostic categories within the section of Anxiety Disorders of the DSM-IV-TR. PTSD and Panic Disorders (PD) according to DSM-IV-TR often involve high levels of physical arousal such as sweating, palpitations, aches and pains, whereas generalized anxiety disorder (GAD) is usually associated with high levels of psychological tension and negative affect (tendencies to worry). From the perspective of the Psychopathology Web, PTSD and PD therefore mostly involve symptoms of the ANXIETY cluster (e.g. autonomic disturbances (it23 and it46) and aches and pains (it24)), whereas GAD borrows its symptoms mainly from the DEPRESSION cluster (e.g. fatigability (it15), concentration difficulties (it16), failing memory (it17), worrying over trifles (it09)). Interestingly, symptoms of PTSD and PD lie closest to the ANXIETY cluster, followed by symptoms of GAD (partly involving items of DEPRESSION), phobic disorders (it11) and OCD (it12) (PSYCHOSIS), which lie on the opposite side of the DEPRESSION cluster relative to symptoms PD and GAD (Figure 3). Hence, rituals and phobias seem to be less directly related to acute anxiety states than symptoms of GAD, PTSD or PD. Indeed, clinical experience dictates that patients with GAD, PTSD and PD are on average more likely to show signs of overt (somatic) anxiety than patients with phobias or OCD. These findings also conform to previous studies on the associations between the various anxiety disorders of the DSM [40]. Future studies could examine the ‘distance’ of neurotic symptoms such as obsessions, phobias, excessive worrying or intrusive memories to symptoms of overt anxiety by calculating the shortest routes that connect these symptoms to the ANXIETY cluster (e.g. using the weighted shortest pathlength measure) [41]. When the (weighted) shortest route between any two symptom in the Psychopathology Web is longer than that between any two other symptoms in the Web, such symptoms are further removed from each other and therefore less related. The validity of this distance measure can be tested by examining how the variance in its scores corresponds to the actual co-occurrence rates of neurotic symptoms and symptoms of ANXIETY.

PSYCHOSIS

The PSYCHOSIS cluster comprised a group of symptoms involving delusions (i.e. ideas of persecution (it31), disrupted thoughts (it30), delusional mood (it33) and other delusions (it36)), hallucinations (i.e. auditory hallucinations (it38), visual hallucinations (it39), commenting voices (it37) and other hallucinations (it40)) and a symptom of feeling controlled (it29). The PSYCHOSIS cluster also comprised the specific neurotic symptoms of depersonalization (it28), derealization (it27) and rituals (it12), which formed direct connections with similar neurotic symptoms within the DEPRESSION cluster (see above). The symptom content of the PSYCHOSIS cluster closely followed that of similar components from the rare complete studies in unselected patient samples. i.e. “Delirium” in [2] and “Paranoid Schizophrenia” in [1]. However, as in the DEPRESSION cluster, the presence of specific neurotic symptoms was unique for the PSYCHOSIS cluster. As discussed, this probably represents the incorporation of symptoms that represent incompletely sampled neurotic syndromes. PCA studies in patients with primary psychotic disorders according to DSM-IV-TR such as schizophrenia have consistently shown a 5-component structure that includes Positive Symptoms (delusions and hallucinations), Negative Symptoms (e.g. affective blunting, slowness of movement, withdrawal), Disorganization (see below), Depression and Excitement / Overactivity [42,43]. The PSYCHOSIS cluster in our study corresponds to the component of Positive symptoms. Remarkably, RETARDATION cluster from our study the closely mimics the Negative Symptoms component in schizophrenic patients, and the BEHAVIORAL DISORGANIZATION cluster resembles the Disorganization component. Also, the DEPRESSION and MANIA clusters from the current study share many similarities with the Depression and Excitement components in psychotic patients (although these components are smaller in psychotic patients than in patients with affective syndromes). Psychosis-like components also appear in groups of patients outside of the category of Schizophrenia. In schizoaffective disorders, Psychosis components alternate or co-occur with affective components of Depression, Anxiety and Excitement [30]. In depressed patients with psychotic features, a Psychosis component emerges with mood-congruent delusions and hallucinations [14,15]. Finally, patients with bipolar disorders often show Psychosis components with delusional mood and delusions of grandeur [14,15,24-27,32]. These findings again suggest that PSYCHOSIS is a universal syndrome that presents itself in different guises depending on patient selection procedures, rating scales and methodologies.

The DSM-5 category of Schizophrenia includes so called A-criteria, which largely conform to the clusters of PSYCHOSIS, RETARDATION and BEH DIS [44]. In DSM-5, increased emphasis is put on the presence of positive symptoms and symptoms of disorganization. The DSM-IV contained a further specification of subtypes of schizophrenia that have been removed from the DSM-5 because of poor validity. Instead, the different manifestations of primary psychotic patients can best be explained by the varying admixtures of principal components or network clusters other than Positive Symptoms / PSYCHOSIS.

RETARDATION

The two remaining network clusters (RETARDATION and BEHAVIORAL DISORGANIZATION) have already been mentioned in relationship to the four network clusters discussed above. In many cases, they emerge as ‘bycatch’ of PCAs in several patient groups. The RETARDATION cluster contained slowness of movement (it60), reduced speech (it54), a lack of appropriate emotional expression (it45), withdrawal from social contact (it49) and moments of sudden absence of awareness or attention amounting to complete unresponsiveness in severe cases (it51). In unselected patient groups, similar components are found using PCA that are referred to as ‘Apathy-Retardation’ [2] and ‘Retardation-Apathy’ [1]. In selected patients groups, a Retardation syndrome emerges in patients with unipolar melancholic depression (as Retardation [7,10,17,18,45]), in patients with Schizophrenia (as Negative symptoms, e.g. [28,46] or Retardation [47]), in patients with bipolar disorders (as Deficit ([25] or Negative Symptoms in [14,27]) in patients with catatonia (as psychomotor slowing [48] or catatonic inhibition [49]) or as side effects of antipsychotic medication (e.g. parkinsonism [50]). Indeed, Retardation is a well-known dimension of psychopathology in clinical research (where it can be separately assessed by the Salpêtrière Retardation Rating Scale [17]), but it is nonetheless often overlooked in clinical practice. Despite the widespread occurrence of symptoms of this dimension across different patient groups and in different areas of psychiatry, it has not got the same self-evident role of an independent syndrome in clinical practice as the 4 previous syndromes. Indeed, the symptoms of RETARDATION do not conform to a separate disease category within the DSM. The common occurrence of symptoms of retardation across different patient groups, and the detection of similar Retardation components using a wide array of different rating scales nonetheless suggests that this component is just as universal as the other four components of psychopathology.

BEHAVIORAL DISORGANIZATION

The BEHAVIORAL DISORGANIZATION (BEH DIS) cluster comprised symptoms of incoherent speech (it57), agitation (it61), distractibility (it48), perseveration (it58) and perplexity (it50). Just like the Retardation component, it is not recognized as a separate universal syndrome in its own right. Nevertheless, it emerges as a separate component in unselected studies of psychopathology (confusingly as ‘Dissociation’ in [2]. In selected patient groups, Disorganization components are consistently found in patients with schizophrenia-spectrum disorders [28,31,51]. These Disorganization dimensions differ somewhat from the DISORGANIZATION cluster of the current study by their accent on cognitive disorganization instead of behavioral disorganization (which may be partially due to the absence of the items of ‘stereotyped thinking’, ‘conceptual disorganization’, and ‘difficulty in abstraction’ in the CPRS). Disorganization components have further been observed in patients with unipolar depression [14,15,19]. Extreme forms of BEH DIS (but not cognitive disorganization) can be observed in patients with manic episodes in bipolar disorders (as Disorganization [14,15], Psychomotor Acceleration [32], or Activation [26]) or in catatonic states (as Involuntary Movements / Manierisms [49]). BEH DIS contains many symptoms commonly observed in Attention Deficit and Hyperactivity Disorder, although few studies have made the comparison [52]. In all, BEH DIS is commonly observed as a part of well-known clinical pictures such as psychosis, mania, catatonia and possibly ADHD. Its consistent emergence across a wide range of patient groups and rating scales again suggests that this syndrome deserves a truly universal status.

Absence of expected clusters

The CPRS lacks items for neurocognitive disorders and uses only two items to measure states of anger, obsessive-compulsive behavior, dissociation and sleeping disorders, whereas phobias and conversion are measured by only a single item each. Such items may represent low-resolution summary states of entire clusters that may yet need to be more adequately sampled. During network community detection, single nodes are not recognized as separate clusters and are incorporated into larger clusters. Only a single item for Obsessive Compulsive Symptoms survived the threshold for significance (it12, rituals). This item was assimilated by the PSYCHOSIS cluster, along with two other items measuring dissociative disorders (it27: derealization and it28: depersonalization). Similarly, items of phobias (it09), conversion (it26) and anger (it04) became part of the DEPRESSION cluster, and items of sleep disorders joined the ANXIETY cluster. Thus, some psychopathological states were sampled rather coarsely by the CPRS, which may have prevented detection of separate clusters for these syndromes. The Psychopathology Web displayed a local region in between the DEPRESSION and PSYCHOSIS clusters that contained relatively many ‘specific’ neurotic symptoms such as worrying over trifles (it09), phobias (it11, including agoraphobia), the obsessive-compulsive symptom of rituals (it12), indecision (it13), and dissociative symptoms such as derealization (it27) and depersonalization (it28). Given the consistent discovery of separate principal components for Anger, Dissociation, Obsessions / Compulsions and Phobias, it seems especially likely that analyses of more richly profiled patients would produce similar network clusters that lie in between PSYCHOSIS and DEPRESSION. The positioning of specific neurotic symptoms in between the major bodies of the PSYCHOSIS and DEPRESSION clusters conforms to previous observations that neurotic syndromes share important features with both PSYCHOSIS and DEPRESSION [53,54]. In clinical practice, such interrelations translate into sometimes difficult-to-make decisions whether to consider certain expressions of OCD as either psychotic or neurotic manifestations (e.g. the idea that the world will end tomorrow if you do not repeatedly count to 100). Similarly, the disproportionally high levels of intention or danger that patients with phobias sometimes attribute to relatively neutral stimuli (such as spiders or heights) may occupy the shady area in between anger, anxiety, psychosis and depression. Dissociation is often seen to overlap with depressive, psychotic and traumatic symptoms and this observation has inspired generations to question the taxonomy of psychiatric illness [55]. In general, neurotic psychopathology may easily slide over into psychotic convictions or states of clinical depression [53]. Future comprehensive rating scales should include a larger number of ‘neurotic’ symptoms to allow for a more fine-grained view of the borderline between affective and psychotic states. A similar argument can be made for the lack of network clusters involving personality domains in the current study. The CPRS provides a rather strict separation between symptoms of psychopathology and personality traits. In most personality rating scales, items do not refer to the phasic states of mental functions (i.e. psychopathology), but long-lived (tonic) traits (e.g. a general tendency to enjoy compliments, explore the environment, or have a negative self-image). Such trait-information is not part of the CPRS, which likely explains the absence of clusters referring to specific domains of personality.

Apart from a lack of items, the absence of some expected clusters may be due to the inability of some clustering techniques to disentangle collections of relatively tightly related items. For instance, the merging of items that measure hallucinations and delusions into a single PSYCHOSIS component is a common finding in multidimensional studies of psychotic symptoms [28,31] and was reproduced in the current study. Nevertheless, one may argue that hallucinations and delusions clearly involve distinct sub-syndromes and that this fact should be reflected in their assignment to different network clusters. In multidimensional studies, such issues are sometimes addressed by performing additional PCAs within the global components, to reveal a microstructure of subcomponents within components. Similarly, we performed network cluster analysis within the six global network clusters (data not shown). This demonstrated the existence of multiple subclusters within each of the six network clusters of the Psychopathology Web. Amongst others, separate subclusters were found within the PSYCHOSIS cluster for 1. hallucinations, 2. paranoid states, 3. obsessive-compulsive and 4. dissociative symptoms, corresponding to the discovery of separate components of positive symptoms using SAPS and SANS rating scales [51]. Also, separate subclusters were found within the ANXIETY cluster for 1. Voluntary (motor) and 2. Involuntary (autonomous) symptoms, which somewhat mimicked the distinction between psychological and somatic anxiety identified in subgroups of anxious patients (see above). Additionally, separate subclusters were found within the DEPRESSION cluster for 1. Hostility and suicide, 2. Decreased interest in food and sex, 3. neurotic symptoms, 4. Remaining items of DEPRESSION. These findings conform to a previous report on the component structure of the MADRS (a rating scale derived from the Depression component of the CPRS) in unipolar depressed patients [56], which showed separate dimensions for Dysphoria, Depression and Vegetative symptoms. The calculation of subclusters requires the disconnection of their corresponding clusters from the remainder of the Psychopathology Web. This caused some loss of information with respect to the relationship of subclusters to the global network structure, hence we chose not to show the details. Nevertheless, such analyses do suggest the presence of an intermediate scale level of organization in between items and clusters that may contain even more elementary syndromes of psychopathology. This may additionally explain the absence of some expected clusters, such as ANGER or OBSESSIVE-COMPULSIVE symptoms. In order to test this hypothesis, we have tested both weighted and unweighted Fast-Greedy network clustering algorithms [57] using our community structure optimization approach, and the MCL clustering algorithm [58]. Weighted and unweighted Fast Greedy algorithms produced clusters that were highly similar to those produced by the Wakita-Tsurumi clustering algorithm (>= 90% similarity with principal components). However, at the optimal pruning threshold for the Wakita clustering algorithm (see Results), the MCL algorithm produces a 11 cluster structure at I = 2.6, scheme =7, in which MANIA, RETARDATION and BEH DIS are perfectly reproduced, DEPRESSION and ANXIETY were slightly confluent at the edges but highly similar to those reported in the current paper and the PSYCHOSIS cluster was reduced into three smaller clusters similar to the subclusters reported above. Also, separate microclusters were found for Dissociation (2 items), Sleeping disorders (2 items), and Rituals (1 item). In all, we found highly similar results with three different clustering algorithms, which is a major encouragement that the clusters reported in the current paper are valid. Different clustering algorithms may indeed produce more or less fine-grained results, although the general cluster structure as reported in the current paper is largely corroborated by other clustering techniques.

Another reason for the absence of some expected clusters in the Psychopathology Web may be the inclusion of too few patients and insufficient power. When the threshold for significance of links was dropped to a level that allowed reattachment of all nodes in the network, an additional network cluster was found in between the major bodies of the MANIA and DEPRESSION clusters that involved observed hostility (it43), labile emotional responses (it44) and disorientation (it52) (data not shown). This suggests the presence of an additional cluster representing reactive moods and dysphoria (e.g. an atypical depression or bipolar mixed factor as in [1]), which was not detected due to a lack of power. Additionally, lowering of the threshold caused a reattachment of it10 (compulsive thoughts) to it12 (rituals) and other neurotic symptoms within the same region. Additionally, it08 (hypochondriasis) reattached to the ANXIETY cluster, it65 (hallucinatory behavior) to the PSYCHOSIS cluster, it47 (sleepiness) to the RETARDATION cluster, it64 (mannerisms and postures) to the MANIA cluster. Thus, the global logic of the Psychopathology Web was spared at lower thresholds.

**References S2**

1. Everitt BS, Gourlay AJ, Kendell RE (1971) An attempt at validation of traditional psychiatric syndromes by cluster analysis. Br J Psychiatry 119: 399-412.

2. Troisfontaines B, Bobon D (1987) [Scales, factor analysis and subscales of the French-language AMDP system]. Acta Psychiatr Belg 87: 23-60.

3. Shafer A (2005) Meta-analysis of the brief psychiatric rating scale factor structure. Psychol Assess 17: 324-335.

4. Bjelland I, Dahl AA, Haug TT, Neckelmann D (2002) The validity of the Hospital Anxiety and Depression Scale. An updated literature review. J Psychosom Res 52: 69-77.

5. Lovibond PF, Lovibond SH (1995) The structure of negative emotional states: comparison of the Depression Anxiety Stress Scales (DASS) with the Beck Depression and Anxiety Inventories. Behav Res Ther 33: 335-343.

6. Shafer AB (2006) Meta-analysis of the factor structures of four depression questionnaires: Beck, CES-D, Hamilton, and Zung. J Clin Psychol 62: 123-146.

7. Goekoop JG, de Winter RP, de Rijk R, Zwinderman KH, Frankhuijzen-Sierevogel A, et al. (2006) Depression with above-normal plasma vasopressin: validation by relations with family history of depression and mixed anxiety and retardation. Psychiatry Res 141: 201-211.

8. Brown TA, Chorpita BF, Korotitsch W, Barlow DH (1997) Psychometric properties of the Depression Anxiety Stress Scales (DASS) in clinical samples. Behav Res Ther 35: 79-89.

9. de Winter RF, Zitman FG, van Houwelingen JC, Wolterbeek R, Goekoop JG (2006) Anxious-retarded depression: relation to two-year outcome of major depressive disorder. J Affect Disord 90: 77-81.

10. Goekoop JG, de BE, Zitman FG (2007) Four-dimensional structure underlying scales for depression anxiety and retardation: emergence of trapped anger and scale improvements. ComprPsychiatry 48: 192-198.

11. Pasquini M, Picardi A, Biondi M, Gaetano P, Morosini P (2004) Relevance of anger and irritability in outpatients with major depressive disorder. Psychopathology 37: 155-160.

12. Bertschy G, Gervasoni N, Favre S, Liberek C, Ragama-Pardos E, et al. (2008) Frequency of dysphoria and mixed states. Psychopathology 41: 187-193.

13. Akiskal HS, Benazzi F, Perugi G, Rihmer Z (2005) Agitated "unipolar" depression re-conceptualized as a depressive mixed state: implications for the antidepressant-suicide controversy. J Affect Disord 85: 245-258.

14. Toomey R, Faraone SV, Simpson JC, Tsuang MT (1998) Negative, positive, and disorganized symptom dimensions in schizophrenia, major depression, and bipolar disorder. J Nerv Ment Dis 186: 470-476.

15. Peralta V, Cuesta MJ, Farre C (1997) Factor structure of symptoms in functional psychoses. Biol Psychiatry 42: 806-815.

16. Bermanzohn PC, Siris SG (1992) Akinesia: a syndrome common to parkinsonism, retarded depression, and negative symptoms of schizophrenia. Compr Psychiatry 33: 221-232.

17. Dantchev N, Widlocher DJ (1998) The measurement of retardation in depression. J Clin Psychiatry 59 Suppl 14: 19-25.

18. Parker G, Hadzi-Pavlovic D, Boyce P, Wilhelm K, Brodaty H, et al. (1990) Classifying depression by mental state signs. Br J Psychiatry 157: 55-65.

19. Parker G, Hadzi-Pavlovic D, Brodaty H, Boyce P, Mitchell P, et al. (1993) Psychomotor disturbance in depression: defining the constructs. J Affect Disord 27: 255-265.

20. Beck AT, Epstein N, Brown G, Steer RA (1988) An inventory for measuring clinical anxiety: psychometric properties. J Consult Clin Psychol 56: 893-897.

21. Keogh E, Reidy J (2000) Exploring the factor structure of the Mood and Anxiety Symptom Questionnaire (MASQ). J Pers Assess 74: 106-125.

22. Shea TL, Tennant A, Pallant JF (2009) Rasch model analysis of the Depression, Anxiety and Stress Scales (DASS). BMC Psychiatry 9: 21.

23. Watson D, Clark LA, Weber K, Assenheimer JS, Strauss ME, et al. (1995) Testing a tripartite model: II. Exploring the symptom structure of anxiety and depression in student, adult, and patient samples. J Abnorm Psychol 104: 15-25.

24. Pacchiarotti I, Nivoli AM, Mazzarini L, Kotzalidis GD, Sani G, et al. (2013) The symptom structure of bipolar acute episodes: in search for the mixing link. J Affect Disord 149: 56-66.

25. Akiskal HS, Azorin JM, Hantouche EG (2003) Proposed multidimensional structure of mania: beyond the euphoric-dysphoric dichotomy. J Affect Disord 73: 7-18.

26. Gonzalez-Pinto A, Ballesteros J, Aldama A, Perez de Heredia JL, Gutierrez M, et al. (2003) Principal components of mania. J Affect Disord 76: 95-102.

27. Lindenmayer JP, Bossie CA, Kujawa M, Zhu Y, Canuso CM (2008) Dimensions of psychosis in patients with bipolar mania as measured by the positive and negative syndrome scale. Psychopathology 41: 264-270.

28. van der Gaag M, Cuijpers A, Hoffman T, Remijsen M, Hijman R, et al. (2006) The five-factor model of the Positive and Negative Syndrome Scale I: confirmatory factor analysis fails to confirm 25 published five-factor solutions. Schizophr Res 85: 273-279.

29. El Yazaji M, Battas O, Agoub M, Moussaoui D, Gutknecht C, et al. (2002) Validity of the depressive dimension extracted from principal component analysis of the PANSS in drug-free patients with schizophrenia. Schizophr Res 56: 121-127.

30. Emsley R, Rabinowitz J, Torreman M (2003) The factor structure for the Positive and Negative Syndrome Scale (PANSS) in recent-onset psychosis. Schizophr Res 61: 47-57.

31. Levine SZ, Rabinowitz J (2007) Revisiting the 5 dimensions of the Positive and Negative Syndrome Scale. J Clin Psychopharmacol 27: 431-436.

32. Cassidy F, Forest K, Murry E, Carroll BJ (1998) A factor analysis of the signs and symptoms of mania. Arch Gen Psychiatry 55: 27-32.

33. Swann AC, Janicak PL, Calabrese JR, Bowden CL, Dilsaver SC, et al. (2001) Structure of mania: depressive, irritable, and psychotic clusters with different retrospectively-assessed course patterns of illness in randomized clinical trial participants. J Affect Disord 67: 123-132.

34. Hantouche EG, Angst J, Akiskal HS (2003) Factor structure of hypomania: interrelationships with cyclothymia and the soft bipolar spectrum. J Affect Disord 73: 39-47.

35. Krabbendam L, Myin-Germeys I, De Graaf R, Vollebergh W, Nolen WA, et al. (2004) Dimensions of depression, mania and psychosis in the general population. Psychol Med 34: 1177-1186.

36. Stein DJ, Fincham D, Seedat S, de Bodinat C, Ahokas A (2009) The DSM-IV-based Generalized Anxiety Disorder Severity Scale: preliminary validation using data from a trial of agomelatine versus placebo. J Nerv Ment Dis 197: 391-394.

37. Elhai JD, Palmieri PA (2011) The factor structure of posttraumatic stress disorder: a literature update, critique of methodology, and agenda for future research. J Anxiety Disord 25: 849-854.

38. Yufik T, Simms LJ (2010) A meta-analytic investigation of the structure of posttraumatic stress disorder symptoms. J Abnorm Psychol 119: 764-776.

39. Jablensky A (2006) Subtyping schizophrenia: implications for genetic research. Mol Psychiatry 11: 815-836.

40. Angst J (1993) Comorbidity of anxiety, phobia, compulsion and depression. Int Clin Psychopharmacol 8 Suppl 1: 21-25.

41. Cramer AO, Waldorp LJ, van der Maas HL, Borsboom D (2010) Comorbidity: a network perspective. Behav Brain Sci 33: 137-150; discussion 150-193.

42. Lehoux C, Gobeil, MH, Lefebvre, AA, Maziade M, Roy, MA (2009) The five-factor structure of the PANSS: a critical review of its consistency across studies. Clinical Schizophrenia and Related Psychoses 3: 103-110.

43. van der Gaag M CA, Hoffman T, Remijsen M, Hijman R, de Haan L, van Meijel B, van Harten PN, Valmaggia L, de Hert M, Wiersma D. (2006 ) The five-factor model of the Positive and Negative Syndrome Scale I: confirmatory factor analysis fails to confirm 25 published five-factor solutions. Schizophr Res 85: 273-279.

44. Tandon R, Gaebel W, Barch DM, Bustillo J, Gur RE, et al. (2013) Definition and description of schizophrenia in the DSM-5. Schizophr Res 150: 3-10.

45. Goekoop JG, Hoeksema T, Knoppert-Van der Klein EA, Klinkhamer RA, Van Gaalen HA, et al. (1992) Multidimensional ordering of psychopathology. A factor-analytic study using the Comprehensive Psychopathological Rating Scale. Acta Psychiatr Scand 86: 306-312.

46. Blanchard JJ, Cohen AS (2006) The structure of negative symptoms within schizophrenia: implications for assessment. Schizophr Bull 32: 238-245.

47. Docx L, Morrens M, Bervoets C, Hulstijn W, Fransen E, et al. (2012) Parsing the components of the psychomotor syndrome in schizophrenia. Acta Psychiatr Scand 126: 256-265.

48. Morrens M, Hulstijn W, Lewi P, Sabbe B (2008) Bleuler revisited: psychomotor slowing in schizophrenia as part of a catatonic symptom cluster. Psychiatry Res 161: 121-125.

49. Kruger S, Bagby RM, Hoffler J, Braunig P (2003) Factor analysis of the catatonia rating scale and catatonic symptom distribution across four diagnostic groups. Compr Psychiatry 44: 472-482.

50. Prosser ES, Csernansky JG, Kaplan J, Thiemann S, Becker TJ, et al. (1987) Depression, parkinsonian symptoms, and negative symptoms in schizophrenics treated with neuroleptics. J Nerv Ment Dis 175: 100-105.

51. Peralta V, Cuesta MJ (1999) Dimensional structure of psychotic symptoms: an item-level analysis of SAPS and SANS symptoms in psychotic disorders. Schizophr Res 38: 13-26.

52. Earnst KS, Kring AM (1997) Construct validity of negative symptoms: an empirical and conceptual review. Clin Psychol Rev 17: 167-189.

53. Kelleher I, Cannon M (2014) Whither the psychosis-neurosis borderline. Schizophr Bull 40: 266-268.

54. Surtees PG, Kendell RE (1979) The hierarchy model of psychiatric symptomatology: an investigation based on present state examination ratings. Br J Psychiatry 135: 438-443.

55. Moskowitz A (2011) Schizophrenia, trauma, dissociation, and scientific revolutions. J Trauma Dissociation 12: 347-357.

56. Suzuki A, Aoshima T, Fukasawa T, Yoshida K, Higuchi H, et al. (2005) A three-factor model of the MADRS in major depressive disorder. Depress Anxiety 21: 95-97.

57. Clauset A, Newman ME, Moore C (2004) Finding community structure in very large networks. Phys Rev E Stat Nonlin Soft Matter Phys 70: 066111.

58. Dongen van S (2000) A cluster algorithm for graphs. Technical Report INS-R0010. Amsterdam: National Research Institute for Mathematics and Computer Science in the Netherlands.
